# Optimizing Omega-3 Polyunsaturated Fatty Acids for Healthy Ageing: Human Intake Evidence and Dairy Cow Dietary Interventions for Milk Enrichment

**DOI:** 10.3390/foods15061079

**Published:** 2026-03-19

**Authors:** Maria Dimopoulou, Panagiotis Madesis, Aliki Dimopoulou, Olga Gortzi

**Affiliations:** 1 Department of Agriculture Crop Production and Rural Environment, School of Agriculture Sciences, University of Thessaly, 38446 Volos, Greece; 2 Department of Psychology, National and Kapodistrian University of Athens, 15784 Athens, Greece; 3 POSS-Driving Innovation in Functional Foods PCC, Sarantaporou 17, 54640 Thessaloniki, Greece

**Keywords:** omega-3 polyunsaturated fatty acids, ageing, dementia, inflammation, cardiovascular disease, milk

## Abstract

As populations around the world continue to age, promoting healthy ageing has become a key public health priority. Nutrition plays a vital role in maintaining physical and cognitive function later in life, and omega-3 polyunsaturated fatty acids (PUFA) are essential components of cell membranes and are known for their anti-inflammatory and cardio-protective effects. Chronic inflammation and oxidative stress are major contributors to age-related decline, and omega-3s help mitigate these processes by modulating immune responses and improving endothelial function. This systematic review aims to examine the potential of omega-3 fatty acids to reduce inflammatory markers and improve overall health. Moreover, it aims to present the most effective dietary interventions in dairy cows that increase PUFA content in milk. PubMed, Web of Science, Scopus, and the Cochrane Library databases were searched for relevant articles published up to November 2025. Evidence suggests that older adults who consume higher levels of PUFA tend to have better cardiovascular health, preserved cognitive function, and a lower risk of age-related diseases such as Alzheimer’s and arthritis, and reduce the risk of frailty and disability in later years. Dietary manipulation to enhance PUFA in bovine milk represents a promising strategy for improving human nutrition while potentially benefiting cow health.

## 1. Introduction

People worldwide are living longer. Today, most people can expect to live into their sixties and beyond [1]. Every country in the world is experiencing growth in both the size and the proportion of older persons in the population. By 2030, one in six people in the world will be aged 60 years or over. At this time, the share of the population aged 60 years and over will increase from 1 billion in 2020 to 1.4 billion [2]. Common conditions in older age include hearing loss, cataracts and refractive errors, back and neck pain, osteoarthritis, chronic obstructive pulmonary disease, diabetes, depression, and dementia. As people age, they are more likely to experience several conditions at the same time [3].

Nutrigenomic convergence is reshaping how we understand food at its deepest level. Food is no longer a passive substrate [4]. It acts as a mechanism of action that guides cellular behaviour and tunes the dynamic dialogue between genome, metabolism, and environment. Each nutrient functions as a signal [5]. Each metabolic step becomes a form of computation. Disease may emerge when ecological responses lose coordination. This shift opens the field of cognitive nutrition. Metabolism becomes a mode of biological rationality. Digestion becomes a sequence of regulated operations. Health becomes the outcome of coherent system-level communication rather than the status of isolated organs [6].

This perspective aligns with the long tradition of scientific thought, where wellness is defined as harmony across the whole organism [7]. When integrated into research, education, and innovation, it positions science in which knowledge, ecology, and medicine form a continuous cultural narrative. Within this view, food becomes a factor contributing to systemic regulatory balance [4]. Oncology may also be viewed as an effort to repair the lost balance between nourishment, biology, and meaning in health, education, and sustainable innovation [8].

From this view, a healthy and balanced diet includes polyunsaturated fatty acids that have been associated with a reduction in the risk of breast and colon cancer [9], as well as inflammation in patients suffering from rheumatoid arthritis [10] and other inflammatory or degenerative diseases [11]. As the dietary omega-3 to omega-6 ratio improves, approaching the ideal ratio of 1:1, the mortality due to heart disease decreases, and this reduction may reach up to 70%. It is vital that omega-3s are in the correct proportions with the other fats. The ideal ratio of omega-3 to omega-6 fats in our diet should be 1:1 [12]. We should have one omega-3 molecule for every omega-6 molecule. However, a ratio of up to 1:5 is also considered a good goal [13]. The ideal ratio of omega-3 to omega-6 fats in our diet should be less than 1:5. However, the ratio of omega-3 to omega-6, recorded in the modern diet, is 1:20 [12].

Moreover, the consumption of fats is essential for the normal functioning of the body. They provide energy, vitamins, and other nutrients, contribute to the absorption of fat-soluble vitamins, and are key factors for the taste of food. However, the type and quantity we consume are of particular importance for health [13]. The quality of fats depends largely on the type of fatty acids they contain (monounsaturated, polyunsaturated, or saturated) [11]. The consumption of monounsaturated and/or polyunsaturated fatty acids (PUFA) is beneficial for health and protects against cardiovascular diseases. Omega-3 fatty acids belong to the family of PUFA, and due to the fact that the human body cannot produce them, they must be ingested through food [14]. Based on this view, enhanced milk with omega-3 fatty acids could be a helpful strategy [15] to increase the consumption without increasing the costs [16]. Flaxseed and camelina are the most effective interventions on cows’ diet, with increases of 30–80% in ALA content in milk fat reported when 5–15% of diet dry matter is replaced by these supplements [17].

This systematic review highlights the potential of omega-3 fatty acids to reduce inflammation markers and improve overall health. Altering the relationship of fatty acids to each other has a negative effect on the body’s ability to manage inflammation, immune function, and hormonal system function [18]. This systematic review fills a literature gap due to the fact that emphasize the dose of omega-3 PUFA with the most beneficial effect on healthy ageing, but also less commonly consumed food sources, such as milk as a strategy with public impact.

## 2. Method

### 2.1. Review Aim and Strategy

This review was performed according to the Preferred Reporting Items for Reviews (PRISMA) 2020 guidelines and checklist (Appendix A) [19]. Moreover, randomized clinical trials are considered the gold standard to demonstrate the dose-effect impact of omega-3 fatty acids on cognitive function, muscle mass, physical function, and overall health [20]. Additionally, this review focuses on the evaluation of the feeding techniques to improve the nutrient profile of milk without loss of organoleptic characteristics and examines their fatty acid content and their impact on overall health. The final search strategy is presented in the Supplementary Materials (Supplementary Table S1).

### 2.2. Literature Search, Study Selection, Eligibility Criteria, and Quality Assessment

This study has inclusive criteria as concern epidemiology data (all age groups, ethnicities, and socio-economic status), design of the studies (controlled trials, but also with emphasis on randomization, variability of the used questionnaires, and sample size), and excluded criteria were the narrative and systematic reviews, studies with limited sizes, and concerns about risk of bias. The risk of bias for included studies was assessed independently by two reviewers (M.D and A.D.) using validated tools appropriate to the study design. For randomized controlled trials (RCTs), the Cochrane Collaboration Risk of Bias tool was applied [21], evaluating domains including the randomization process, deviations from intended interventions, missing outcome data, outcome measurement, and selective reporting. For non-randomized or observational studies, the Newcastle–Ottawa Scale (NOS) was used, assessing selection, comparability, and outcome/exposure domains. Each study was rated as low, moderate/some concerns, or high risk of bias according to the guidance of the respective tool. Disagreements between reviewers were resolved through discussion or consultation with a third reviewer. Studies were excluded if they met any of the following predefined criteria: rated as high risk of bias in one or more critical domains for the Cochrane Collaboration Risk of Bias tool, or scored below [insert threshold, e.g., <5 out of 9 stars] on the Newcastle–Ottawa Scale, indicating poor methodological quality, or demonstrated significant methodological limitations likely to substantially affect outcome validity (e.g., severe attrition bias > 30%, lack of control group, or inadequate reporting of primary outcomes). Only studies meeting the predefined quality threshold (low risk or moderate risk without critical flaws) were retained for qualitative and/or quantitative synthesis. As for the second section is a narrative review. A comprehensive search encompassing three databases was conducted as follows: (1) The search on PubMed and Scopus involved the utilization of MeSH terms specifically focusing on “# Health,” AND/OR “#Diet,” AND/OR “#Polyunsaturated fatty acids,” AND/OR “#Intervention’’, “#Randomized clinical trial,” AND/OR “#Older adults,” AND/OR “#Ageing,” AND/OR “#Cognitive function,” AND/OR “#Muscle Mass”, AND/OR “#Physical Function”, “#Strength,” AND/OR “#Inflammation,” AND/OR “#Dairy Cow,” AND/OR “#Nutritional profile,” AND/OR “#Milk”. The aim was to investigate the relationship between the dose of omega-3 PUFA and their impact on overall health, as well as the impact on the nutritional profile of milk due to the cow diet interventions. The preliminary search produced 202 articles, with 57 duplicates excluded, resulting in 145 articles for further analysis. (2) The search strategy for the Web of Science database concentrated on identifying articles related to “# Health,” AND/OR “#Diet,” AND/OR “#Omega-3 polyunsaturated fatty acids,” AND/OR “#Intervention’’, AND/OR “#Randomized clinical trial,” AND/OR “#Older adults,” AND/OR “#Ageing,” AND/OR “#Cognitive function,” AND/OR “#Muscle mass”, AND/OR “#Physical function”, AND/OR “#Strength,” AND/OR “#Inflammation.” AND/OR “#Mental health,” AND/OR “#Exercise,” AND/OR “#Lifestyle,” AND/OR “#Cardiovascular disease,” AND/OR “#Cancer”, AND/OR “#Dairy Cow,” AND/OR “#Nutritional profile,” AND/OR “#Milk”. Other terms such as “#Food” AND/OR “#Nutrition” were also included to ensure a broader scope. The objective was to explore dietary habits, nutritional interventions, and the role of various diet patterns in modulating specific bioactive compounds and addressing related disorders. The initial search yielded 5 articles, and after eliminating 3 duplicates, 2 articles remained for detailed scrutiny. (3) For the Cochrane Library search, emphasis was applied to #Omega-3 polyunsaturated fatty acids, AND/OR #Interventions, AND/OR #Nutrition, AND/OR #Overall health, AND/OR #Randomized clinical trials, with an additional keyword “#Supplementation. The primary aim was to identify articles examining the association between omega-3 polyunsaturated fatty acids consumption and overall, as well as mental health, with particular attention to the guidance provided by health professionals. Abstracts were screened using Rayyan. If there was any doubt about the inclusion of an article, this was included for full-text review. Full articles were screened independently by two authors using Rayyan. Disagreements were resolved by returning to the original article along with a third senior author (O.G.) when required.

**Figure 1:**
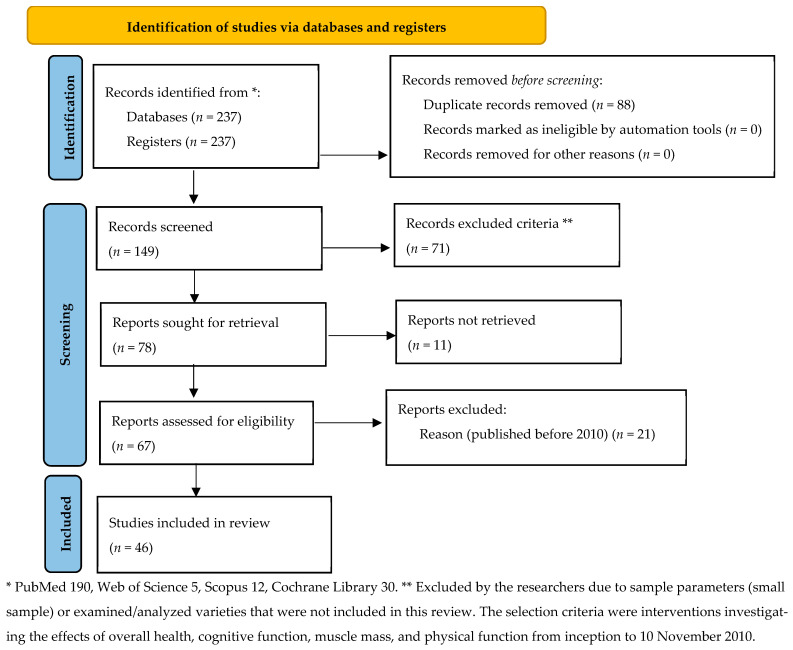
Prisma flow chart of the study (version 2000) as follows: randomized clinical trials of long-chain omega-3 PUFA in older adults [22,23,24,25,26,27,28,29,30,31,32,33,34,35,36,37,38,39,40,41,42,43,44,45,46,47,48,49,50] and interventions in dairy cows’ diets to increase omega-3 unsaturated fatty acids [51,52,53,54,55,56,57,58,59,60,61,62,63,64,65,66,67]).

One investigator (M.D.) screened all of the included studies for the risk of bias, with a separate investigator (A.D.) independently validating the risk of bias. The Cochrane risk of bias tool was used for randomized controlled trials [21]. The selection of these databases was based on their reputability, comprehensiveness, and relevance to healthcare and medical research (Table 1). The inclusion and exclusion criteria are shown in Table 2. The studies included in this review are shown in Figure 1.

## 3. Results

### 3.1. Omega-3 Health Benefits

Omega-3s support cardiovascular health, brain function, and efforts to live a long, healthy life. Given the difficulty of obtaining adequate omega-3s from diet alone (i.e., from fatty fish, sea vegetables, and other omega-3-rich foods), and how far-ranging the benefits of omega-3s are, many Americans take a daily omega-3 supplement. According to a 2016 study, women over 50 might want to consider upping their dose even more to promote cognitive vitality as they age, but many consumers do not get enough omega-3 fatty acids (and their benefits) from current fish oil supplements [22].

#### 3.1.1. Omega-3s Bolster Cognitive Function

In the double-blind, placebo-controlled study, cognitively healthy adults between the ages of 60 and 80 that took a high-potency dose (2.3 g) of long-chain polyunsaturated omega-3 fatty acids (i.e., EPA and DHA) in supplement form as fish oil for 18 months presented no significant changes [23] but the same supplementation (430 mg DHA and 90 mg EPA) for 24 weeks demonstrated increased omega-3 index levels in participants (i.e., the biomarker for omega-3 status in your body) and significantly enhanced memory recall compared to the control group [22]. This may be due to the longer duration of the second trial [22]. This suggests that taking a high-potency supplement with marine omega-3s EPA and DHA can help maintain cognitive function and support memory across the lifespan. According to this systematic review, these brain-critical nutrients support other aspects of cognition as well, including learning, cerebral blood flow, and even mood support [24,26,27,28,29,30,31,68] (Table 3), and omega-3 fatty acids can help you age gracefully in other ways, too [22].

#### 3.1.2. Functional Nutrition Training

Food may be used as a therapeutic tool with a cutting-edge nutrition deep dive taught by the health and wellness experts. PUFA supplementation could play a crucial role in improving muscle anabolic response and strength in older adults [32,33,34,35,36,37,38].

#### 3.1.3. Omega-3s Support Longevity

Evidence suggests that omega-3s help us live a long, healthy life by supporting not only memory and cognitive function, but also vision longevity, joint mobility [32,33,34,35,36,37,38], heart health [40], antioxidant activity, and social well-being as well. Not all omega-3 supplements are of equivalent quality. The effectiveness and safety of an omega-3 product can vary widely depending on several factors, including the source of the oil, purity, concentration, form of the fatty acids, and manufacturing standards. Consumers need to look for a sustainably sourced fish oil with a clinically meaningful dose of marine omega-3s to reap the myriad longevity benefits these healthy fats have to offer [50]. High-potency omega-3 supplementation can help people over 50 maintain cognitive function, enhance memory recall, and support overall brain longevity. Furthermore, omega-3s have been shown to help bolster other facets of health as we age (e.g., vision, joint mobility, heart health, antioxidant status). Adding a high-quality fish oil supplement to older adults’ daily routines could ensure brain [24,26,27,28,29,30,31,68] and body health [40,41,42,43,44,45,46,47,48].

**Figure 2:**
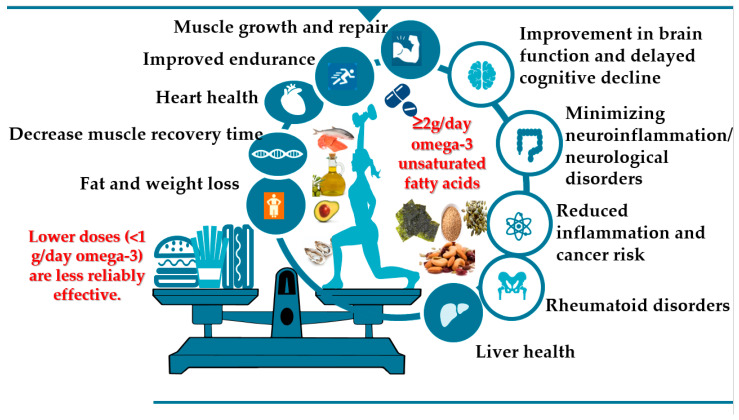
Beneficial effect of omega-3 unsaturated fatty acids consumption to overall health (based on Table 3 and Berglin et al., Scandinavian J Rheumat, 2013) [10,22,23,24,25,26,27,28,29,30,31,32,33,34,35,36,37,38,39,40,41,42,43,44,45,46,47,48,49,50].

Higher doses of omega-3 fatty acids (in the multi-gram range, e.g., ~3.3 g/day) and longer durations (≥12 months) are associated with more consistent positive outcomes (in cognition, in one trial). Lower doses (<1 g/day) and shorter durations (~6 months) often yield no significant effect (Figure 2). For muscle/physical outcomes: some modest benefit shown in meta-analysis with doses up to ~3.36 g/day, but in trials with lower doses, the effect is minimal or non-significant. Thus, in patients who are not in the early post myocardial infarction setting, high-dose marine omega-3s (4 g per day) may be required to provide cardiovascular benefit when given on top of optimized medical therapy [69]. Trials/meta-analyses indicate that benefits are more likely if baseline DHA and EPA levels are not already very high; i.e., there is more “room for improvement” [70].

Regarding cognition, ~3.36 g/day × 30 months improves multiple cognitive domains in a specific at-risk older population [24]. Smaller doses (≈500–1000 mg/day) show possible benefit (especially executive function), but the effect size is modest and inconsistent. For better muscle/physical performance, doses ranging from 0.7 g to 3.36 g/day show some benefit in physical performance in older adults; muscle strength gains are less consistent, especially at lower doses [24,71]. Low doses (<1 g/day) in older adults with normal/mild impairment (e.g., ~400–600 mg/day) over ~6 months did not show meaningful benefit [68]. In healthy ageing, the brain becomes less responsive to nutritional signals due to the reduced DHA transport efficiency, increased oxidative stress, chronic low-grade inflammation, and altered lipid metabolism, so older adults often need higher doses to achieve the same brain DHA levels as younger adults. Moreover, at lower intakes, much of the omega-3 is used for systemic functions (cell membranes, inflammation modulation) rather than deposited in neural tissue. Only at higher doses does enough accumulate in neuronal membranes to meaningfully alter synaptic plasticity, membrane fluidity, and neurotransmission [24,71].

If aiming for an effect in older adults for healthy ageing domains (cognition or physical function), evidence suggests a dose in the range of ~2–3 g/day of combined EPA and DHA over ≥12 months may increase the likelihood of benefit [22,38]. Doses much below ~1 g/day appear unreliable for meaningful effect in trials, at least over shorter durations. However, a higher dose does not automatically guarantee benefit—other factors (baseline status, diet, health status, duration, and concurrent lifestyle) also matter [70]. Improvement in lipid biomarkers has also mentioned in two studies [41,72], and two other studies found sex differences and women had the most beneficial effect of omega-3 fatty acids consumption [39,73]. Moreover, multi-nutrient supplementation (1 g DHA, 160 mg EPA, 240 mg ginkgo biloba, 60 mg phosphatidylserine, 20 mg d-α tocopherol, 1 mg folic acid, and 20 µg vitamin B12) improved cognition and mobility in able older females at clinically relevant levels, suggesting a potential role in reducing the decline to frailty [74].

**Figure 3:**
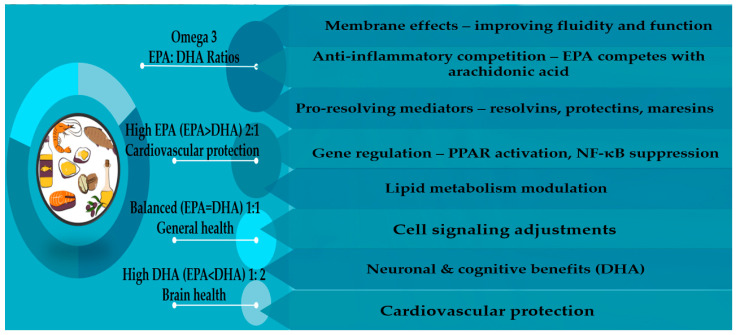
Possible mechanism of action of omega-3 unsaturated fatty acids and the most beneficial ratios of EPA and DHA according to Siddiqui et al. (Mini reviews in medicinal chemistry, 2004), Frumuzachi et al. (Antioxidants, 2024), Bays et al. (Expert Rev Cardiovasc Ther, 2008), and Shibabaw et al. (Mol Cell Biochem, 2021) [75,76,77,78].

The mechanism of action is not fully understood, but the most plausible mechanisms include the following: (1) Omega-3s become part of the phospholipid bilayer of cell membranes and increase membrane fluidity, improve membrane receptor function (e.g., insulin receptors, immune receptors), and improve ion channel activity (important in neurons and cardiac cells) [75]. (2) EPA-derived eicosanoids (PGE_3_, LTB_5_) are less inflammatory, less vasoconstrictive, and less platelet-aggregating than AA-derived ones (PGE_2_, LTB_4_), so they cause a weaker inflammatory response. (3) Omega-3s minimize the expression of inflammatory cytokines and lipogenesis (fat synthesis), increase fat oxidation, production of antioxidant enzymes, and improve glucose and lipid metabolism [76]. (4) They reduce activation of NF-κB, reduce inflammatory gene expression, and interfere with Toll-like receptor 4 (TLR4) signalling modulation of mTOR, MAPK, and PI3K/Akt pathways. (5) Additionally, omega-3s support synaptic function, modulate neurotransmission, promote neurogenesis, affect plasticity, protect against oxidative stress in neural tissues, modify G-protein–coupled receptor 120 (GPR120), and so increase anti-inflammatory signalling. (6) EPA and DHA alter enzymes and pathways involved in lipid handling, such as reduced triglyceride synthesis in the liver, increased fatty acid β-oxidation, changes in LDL particle size, and improved membrane composition in lipoproteins [77]. (7) Finally, omega-3s affect the cardiovascular system through decreased platelet aggregation (EPA derivative mediators are less pro-aggregatory), triglycerides, anti-arrhythmic membrane-stabilizing effects, improved endothelial function via NO production, and reduced vascular inflammation (Figure 3) [78].

### 3.2. Interventions in Dairy Cows’ Diets to Increase Omega-3 Unsaturated Fatty Acids

Compared with fish-based solutions, dairy-based enrichment is more scalable, reduces pressure on marine resources, and integrates into existing agricultural systems. That is very important for public health nutrition strategies [51]. Dairy cow dietary intervention was chosen as a scalable, sustainable strategy to enhance population-wide omega-3 PUFA intake through habitual foods, and rumen-protected marine supplements proved most effective at increasing cognitively relevant DHA and EPA levels in milk [52]. The “best” supplementation balanced: magnitude of omega-3 increases, milk yield and fat depression risk, oxidative stability of milk, animal health and palatability, cost, and scalability. Rumen-protected marine omega-3 sources, possibly combined with plant-based ALA sources, provide the optimal compromise. In addition, ALA conversion to DHA [18] in humans is low but still contributes to overall omega-3 status [52]. Typical bovine milk contains relatively low levels of these long-chain omega-3 PUFA [18], mainly because of extensive biohydrogenation in the rumen, which saturates unsaturated fats [51]. Therefore, nutritionists have studied dietary strategies to enrich milk with omega-3 PUFA by (a) supplying rich omega-3 sources and (b) protecting them from ruminal transformation [52].

Feeding fresh pasture is a classical and natural way to boost ALA in milk, because leafy forages (grasses, legumes) are naturally rich in ALA [66]. While not always quantified in intervention trials, grazing systems tend to yield milk with a more favourable omega-6: omega-3 ratio compared to total mixed ration (TMR) feeding systems [53]. The most commonly used plant source is flaxseed (linseed) and its oil, because of its high ALA content. In Friesian dairy cows, the addition of whole flaxseed to the diet (versus control) significantly increased omega-3 PUFA in milk, including ALA (with beneficial shifts in milk saturated fat, monounsaturated fat, and conjugated linoleic acid, CLA) [17]. A recent study found that feeding 250 g/d or 500 g/d of flaxseed in a TMR did not compromise dry matter intake or milk yield, yet significantly raised milk omega-3 content and reduced the omega-6: omega-3 ratio [54]. Křížová et al. (2022) compared whole flaxseed with ground flaxseed and showed that both increased total omega-3 PUFA in plasma and milk, but GF led to a greater increase in ALA yield in milk, and a higher milk ALA/ALA intake ratio [55]. Different flaxseed forms also influence rumen fermentation and microbial ecology. A study comparing whole vs. ground flaxseed found that ground flaxseed led to higher proportions of volatile fatty acids (acetate, propionate, etc.) and altered the rumen bacterial community, suggesting that processing affects not just lipid supply but ruminal metabolism [56].

Rumen-protected tuna oil (EPA/DHA source) has been shown to dramatically increase EPA and DHA in milk fat. In an early study, supplementation with rumen-protected tuna oil caused a 3–4-fold increase in total omega-3 PUFA, without negative effects on milk yield, fat, protein, or sensory characteristics [53]. More recently, studies have used rumen-protected microalgae powder. For example, supplementing Holstein cows with coated *Shizochytrium* algae (a DHA-rich microalgal form) significantly increased plasma DHA and milk DHA, with a higher transfer rate of DHA to milk than uncoated forms [57]. Saadaoui et al. (2021) also reviewed microalgae supplementation, noting that *Schizochytrium* and *Nannochloropsis* are among the most effective strains, with robust increases in DHA and EPA in milk, without compromising the oxidative stability of the milk [51]. Because the rumen hydrogenates many unsaturated fatty acids, simply feeding high-ALA or DHA sources is often not enough—the fats must be “protected” to improve their transfer to milk [58]. Rumen-protected fat (RPF) technologies and common methods include calcium salts of long-chain fatty acids, microencapsulation, pH-sensitive coatings, and formaldehyde-treated encapsulation [59]. These reduce lipolysis and biohydrogenation in the rumen, increasing the proportion of dietary omega-3 that reaches the small intestine and ultimately the mammary gland [51]. Altering the basal diet can help [58], for instance, increasing the omega-3:omega-6 ratio by replacing extruded soybeans (rich in n-6) with extruded flaxseed led to beneficial microbial shifts in the rumen and reduced indicators of inflammation in transition cows [60]. By promoting specific rumen bacterial populations and reducing lipopolysaccharide (LPS) translocation, this strategy may both improve milk fatty acid profile and support cow health [58].

Flaxseed (especially ground) consistently raises milk ALA [55]. Isenberg et al. (2025) reported an increase in milk ALA and total omega-3 PUFA in supplemented cows [61]. Marine oils (like protected tuna oil) and microalgae significantly boost milk EPA and DHA. For example, the coated Schizochytrium supplement improved milk DHA levels and had a higher transfer rate compared to uncoated forms [60]. Many of the interventions reduce the omega-6:omega-3 ratio of milk, which is nutritionally desirable for human consumption [65]. For instance, flaxseed supplementation not only increases omega-3 but can lower this ratio, as shown in the 2025 flaxseed dose–response study [54].

Flaxseed at moderate levels generally does not adversely affect milk yield or basic milk composition [54]. In the 2025 study, even with 500 g/day flaxseed, there was no negative effect on milk fat or yield. While some early days of feeding coated microalgae showed a temporary drop in milk fat, fat content tended to recover over time [57]. There may also be positive systemic effects. For instance, supplementing with omega-3 PUFA has been associated with immune and reproductive benefits (larger corpus luteum, higher progesterone) [62]. The rumen microbial community plays a critical role in how dietary fatty acids are processed. Recent research highlights that replacing high n-6 sources (e.g., soy) with flaxseed (high omega-3) can reduce the relative abundance of Gram-negative bacteria, lower plasma LPS, and down-regulate inflammatory pathways (e.g., NF-κB) in the liver, especially in transition cows [60]. Different forms of flaxseed (whole vs. ground) alter ruminal fermentation patterns and VFA production. Ground flaxseed increases propionate and other VFAs, perhaps via shifts in bacterial fermentation [56]. When designing interventions, practical constraints must be considered, such as the cost, rumen health, processing of seeds, and consumer demand. Protected marine oils or microalgae (especially coated) are more expensive than plant oils or seeds. High levels of unprotected unsaturated fats may impair fibre digestion or cause milk fat depression, so balance is required [63]. Grinding or extrusion increases ruminal availability, but unprotected fats may get biohydrogenated; protected forms are ideal. Markets for “omega-3-enriched milk” may justify higher feeding costs, but producers need to ensure a consistent supply and profitability [79]. Expected changes in milk fatty acid composition with diet interventions: pasture, flaxseed, linseed oil, fish oil, rumen-protected fats are summarized in Table 4, Table 5 and Table 6 [51,52,53,54,55,56,57,58,59,60,61,62,63,64,65,66,67,80].

**Figure 4:**
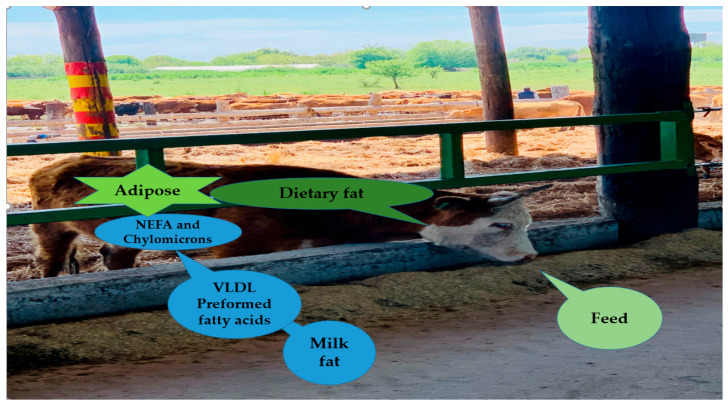
Make better nutritional and management decisions by understanding milk fatty acids mixed and preformed synthesis according to Suksombat et al. (Asian-Australas J Anim Sci, 2013) [64].

Certain supplements (fish oil, high PUFA oils) can depress milk fat if fibre is low or rumen pH drops; proper ration balancing prevents this [80]. Milk fatty acids come from two main sources: (1) de novo (and mixed) synthesis in the mammary gland, and (2) preformed fatty acids taken directly from the cow’s diet or body fat. Knowing the difference is critical because each source responds differently to nutrition, health, and management. Mentioning mixed and preformed milk fatty acid synthesis is important because it connects nutrition directly to milk composition, provides insight into rumen health and energy balance, and enables proactive, data-driven feeding and management decisions (Figure 4).

According to a recent study, herds with healthy rumen fermentation and low risk factors for milk fat depression will have de novo fatty acids of 0.87 g/100 g milk, mixed-origin fatty acids of 1.41 g/100 g milk, and preformed fatty acids of 1.46 g/100 g milk [67].

## 4. Discussion

A growing body of evidence supports a beneficial role of omega-3 PUFA—particularly EPA (eicosapentaenoic acid) and DHA (docosahexaenoic acid)—in promoting healthy ageing across multiple physiological systems [81]. Their overall health effects have been documented from many studies according to this systematic review and more specifically to cardiovascular health, cognitive function and brain health, inflammation and immune function, and musculoskeletal health (Table 1 and Table 2). Twenty-nine randomized controlled trials (RCTs) included in this systematic review (Figure 1) indicate that omega-3 intake lowers triglyceride levels, reduces blood pressure slightly, and improves endothelial function. These effects collectively decrease the risk of atherosclerosis and cardiovascular mortality- key determinants of longevity and overall longevity and function (Table 3). Across RCTs, omega-3 supplementation shows modest, dose-dependent benefits for healthy ageing in older adults, mainly through improvements in cognitive function and muscle health at higher intakes (2–4 g/day EPA and DHA) over prolonged periods (≥6–12 months) [22,24,31]. The effects are not universal; they depend on baseline omega-3 status, health condition, and concurrent lifestyle interventions. RCTs consistently show that higher doses (≥2 g/day) of EPA + DHA and longer durations (≥12 months) are needed to elicit measurable benefits in cognition, muscle mass, or function [22,24]. Lower-dose trials (<1 g/day) generally report null results [68]. Evidence from long-term, high-dose RCTs indicates modest cognitive improvements, especially in executive and verbal domains. However, smaller RCTs show substantial heterogeneity, suggesting benefits are domain-specific and population-dependent (e.g., low baseline omega-3 status or vascular disease). High-dose fish-oil supplementation (≥2–4 g/day) enhances muscle anabolic signalling and volume, particularly when combined with resistance training [32,33,34,35,36,37,38]. In sedentary or low-dose settings, results are minimal [33]. Large RCTs like VITAL confirm cardiovascular safety and modest protection, but do not provide clear effects on overall physical or cognitive ageing trajectories [40].

Overall, omega-3 fatty acids represent a safe, biologically plausible, and supportive strategy for promoting aspects of healthy ageing (Figure 3), but they are adjuncts, not substitutes, for comprehensive dietary and physical activity measures [32,33,34,35,36,37,38]. Furthermore, large, long-duration RCTs are warranted to clarify optimal dose, target populations, and clinically meaningful outcomes [82].

DHA is a major structural component of neuronal membranes. Some studies associate higher omega-3 levels with slower cognitive decline and a lower risk of Alzheimer’s disease [83]. Some RCTs show modest improvements in memory and executive function, particularly in individuals with low baseline omega-3 status. A dose–response meta-analysis (up to December 2024) of 58 RCTs found that each +2000 mg/day (i.e., 2 g/day) of omega-3 supplementation was associated with improvements in various cognitive domains (attention, perceptual speed, language, etc.) though the certainty of evidence was low to moderate [84], and the results of this systematic review are in totally agreement. Another older meta-analysis reported that RCTs with doses in the range 400–1800 mg/day (i.e., 0.4–1.8 g/day) (DHA and EPA) produced a small statistical improvement in MMSE (cognition) in older adults [85]. However, a very large meta-analysis found no clear effect of long-chain omega-3 on new neurocognitive illness or global cognition, and they found no consistent differential effect by dose or duration [86]. In one RCT in older adults with coronary-artery disease, a high dose of ~3.36 g/day EPA and DHA over 30 months improved cognitive tests compared to control [87]. Effects of supplementation with omega-3 polyunsaturated fatty acids on cognitive performance and cardio metabolic risk markers in healthy 51- to 72-year-old subjects: a randomized controlled cross-over study (2012). Although older, this RCT showed a 5-week daily intake of fish-oil omega-3 improved some cognitive tests and cardio metabolic risk markers in healthy middle-aged to older adults [88]. Early RCT evidence hinted at cognitive and cardio-metabolic benefits, though small and short-term. Cognitive benefits may appear in the range ~1–3 g/day of EPA and DHA, but results are inconsistent; higher doses (e.g., ~3 g/day) may show benefit in specific populations (e.g., older adults with comorbidity). There is no firmly established “ideal” dose for all.

Ageing is accompanied by chronic, low-grade inflammation. Omega-3 PUFA modulate inflammatory pathways by reducing pro-inflammatory cytokines and promoting the production of anti-inflammatory lipid mediators (resolvins, protectins) [81]. This contributes to reduced tissue damage and better immune regulation [89]. In a prospective cohort of older adults, higher circulating levels of long-chain omega-3 PUFA (from seafood: EPA, DPA, DHA) were associated with an ≈18% lower risk of “unhealthy ageing” (defined as survival without major chronic disease and intact cognitive and physical function after age 65) [90]. Individually, EPA (≈15% lower risk) and DPA (≈16% lower risk) showed significant associations; DHA did not show a statistically significant association in that study [90]. Higher long-chain omega-3 in blood may reflect better “healthy ageing” outcomes, though this is observational (an association, not proof of causation according to the European Food Safety Authority [91]).

Emerging studies suggest that omega-3s may enhance muscle protein synthesis, improve muscle strength, and reduce sarcopenia risk in older adults, especially when combined with resistance exercise and adequate protein intake. A meta-analysis of older adults found that when supplementation exceeded 2 g/day of omega-3 PUFA, and a duration of ≥6 months, there was some benefit, e.g., muscle mass gain ~0.67 kg (95% CI 0.16–1.18 kg) and improved walking speed [92]. A more recent review found that omega-3 supplementation in older adults had no significant effect on muscle mass or function, but a very small effect on muscle strength (SMD = 0.12), and that subgroup analyses did not show that dose (<2 vs. ≥2 g/day) or age significantly changed the outcome [93]. In studies combining supplementation and resistance training, there was no clear added benefit of the omega-3 component; the dose-effect was unclear [94]. Meta-analysis of 10 RCTs’ findings emphasizes a small increase in muscle mass (~0.33 kg; 95%CI: 0.05–0.62) and improvement in timed up and go performance (−0.30 s; 95%CI: −0.43 to −0.17) with omega-3 supplementation in older adults, a ≥2 g/day dose and ≥6 months’ duration showed larger effects. The evidence suggests some benefit of omega-3 for preserving/improving muscle performance in older adults, though effects are modest [92]. For muscle/mobility outcomes in older adults, doses of ≥2 g/day of EPA and DHA show modest improvements in some studies, especially long-term (≥6 months). But evidence is mixed, and effects are small [32,33,34,35,36,37,38].

Cohort studies link higher blood levels of omega-3s with lower all-cause mortality and longer telomere length, suggesting a possible role in cellular ageing and lifespan extension [95]. Omega-3s exert their influence through anti-inflammatory, neuroprotective, and membrane-stabilizing mechanisms, improving neuronal signalling, vascular function, and mitochondrial efficiency, all of which are crucial in slowing age-related decline. Overall, 1200 mg/day of EPA and DHA may help to reduce CRP concentration in patients with cardio-metabolic disorders. This reduction is clinically significant, and thus intervention with omega-3 fatty acids may be considered for this population [96].

Summarizing, omega-3 PUFA contribute to healthy ageing through cardiovascular protection, neurocognitive preservation, anti-inflammatory effects, and maintenance of muscle function. While dietary intake via fatty fish remains optimal, supplementation can be beneficial, particularly for individuals with low omega-3 consumption (Figure 2) [88]. However, findings from intervention trials remain heterogeneous, and more large-scale, long-term RCTs are needed to establish causal relationships [82]. Suggested practical dose ranges (based on current evidence) for cognition/brain health: ~1 g/day (1000 mg) to ~3 g/day EPA + DHA seems supported in some RCTs (e.g., ~3.36 g/day in one trial). For muscle/mobility: ≥2 g/day may be beneficial, particularly if sustained for ~6+ months. Lower doses (<1 g/day) appear to have little consistent effect in RCTs, e.g., one study found that low-dose omega-3 did not significantly improve cognitive status among older people [25]. These doses refer to combined EPA and DHA (long-chain omega-3) unless otherwise specified. For older adults aiming to support healthy ageing (cognition, muscle/mobility), supplementation of EPA and DHA in the range of approximately 1 g/day to 3 g/day appears supported by some evidence; for muscle/mobility benefits, targeting ≥ 2 g/day for at least 6 months may increase chances of seeing benefits; lower doses (<1 g/day) are less reliably effective [34,35,36,38,39]. Supplementation needs to always be paired with a good diet, physical activity (particularly resistance exercise), adequate protein, and medical supervision [39].

These doses are derived from clinical trials in older adults; they do not guarantee benefits, and results vary by population (healthy or at-risk), baseline omega-3 status, diet, duration of supplementation, and concurrent lifestyle factors (exercise, protein intake, etc.) [97]. Higher doses may carry a higher risk of side effects (e.g., bleeding risk, gastrointestinal upset), especially in people on anticoagulants or with certain health conditions—a physician should always be consulted [98]. Many trials do not standardize dose by body weight; older adults with higher body mass may require higher doses for the same effect. For muscle/mobility outcomes, the effect of omega-3 appears small and may be enhanced by resistance training, sufficient protein, and other lifestyle measures. Supplementation does not replace a healthy diet (especially fatty fish intake) [99] and other lifestyle elements (exercise, sleep, etc.) [100]. Long-term safety data at high doses (>4 g/day) are less abundant in older, frail populations. Some meta-analyses show no dose-effect or unclear dose–response (especially in cognition), so more research is still needed due to the fact that one review reported “no significant associations between treatment duration or dosage and cognitive outcomes” [101].

Omega-3 fatty acids (especially DHA and EPA) mainly influence neuronal membrane fluidity, synaptic signalling, inflammation reduction, and cerebral blood flow [56,57], so according to most studies, individuals are more likely to see improvements in memory and learning, attention, and mood-related cognition, with a smaller effect on complex executive functions, which are long-established cognitive decline skills that depend heavily on practice or education [22,23,24,25,26,27,28,29,30,31]. Omega-3 polyunsaturated fatty acids play a significant role in supporting healthy ageing by protecting cardiovascular and cognitive health, reducing inflammation, and maintaining physical function [102]. Incorporating omega-3-rich foods into the diet represents a simple yet powerful strategy to promote longevity and quality of life in older adults [99]. The World Health Organization and numerous health agencies recommend at least two servings of oily fish per week for adults, with possible supplementation in populations with low dietary intake [103]. Older adults with sarcopenia may benefit from at least three servings per week of fish in order to have a minimum intake of 4–4.59 g daily of omega-3, and reach the 50% RDA in Vitamin E and D [104].

The bioavailability of omega-3 fatty acids—primarily EPA and DHA—depends on their chemical form, the food matrix, and individual digestive factors. Omega-3s are absorbed more efficiently when consumed as triglycerides or phospholipids (as in fish and krill oil) compared with ethyl-ester forms commonly found in some supplements, which require additional enzymatic processing [105]. Taking omega-3 with a meal containing dietary fat significantly enhances absorption by stimulating bile release and micelle formation. Other influences include the presence of competing fatty acids, overall gut health, and individual metabolic differences. Because of these variables, whole-food sources such as fatty fish generally provide better and more consistent bioavailability than many supplemental forms [106].

Nowadays, dietary manipulation to enhance omega-3 PUFA in bovine milk is a promising strategy for improving human nutrition while potentially benefiting cow health [58]. This systematic review demonstrates that both forage-based and supplemental interventions can increase milk ALA, EPA, and DHA (Figure 4), but the efficiency of transfer to milk depends on the source, form, and protection of fatty acids against ruminal biohydrogenation (Table 4, Table 5 and Table 6). Forage and pasture-based feeding consistently results in higher milk omega-3 content compared to total mixed ration (TMR) systems, largely due to the high ALA content of fresh leafy forages [51]. However, pasture availability is seasonal and geographically dependent, and its contribution to omega-3 enrichment can be limited in indoor or feedlot systems. These constraints have prompted the exploration of oilseed supplements, particularly flaxseed, which reliably elevates milk ALA concentrations. Processing methods, such as grinding or extrusion, influence both bioavailability and rumen fermentation patterns, with ground flaxseed often providing greater ALA transfer efficiency but also modulating volatile fatty acid (VFA) profiles and microbial populations [107]. Marine and microalgae sources provide direct EPA and DHA, which are otherwise present in low concentrations in milk. Coated or rumen-protected forms of microalgae and fish oils substantially improve milk omega-3 content without adversely affecting milk yield or fat content [60]. These interventions, however, are often limited by higher cost and availability, emphasizing the need for cost-effective protection technologies.

Rumen biohydrogenation remains the primary physiological barrier to effective omega-3 enrichment. Strategies that combine rumen-protected lipids with diet composition adjustments—such as higher forage-to-concentrate ratios or selective inclusion of tannins and essential oils—show promise in limiting microbial saturation of unsaturated fatty acids [51]. Such approaches not only enhance the milk fatty acid profile but may also positively influence systemic inflammation and metabolic health in cows [80].

Twenty-nine studies included in this review suggest that moderate supplementation of omega-3 sources, when adequately protected, can improve milk fatty acid composition without negative impacts on lactation performance [108]. Variation in milk fat responses to nutritional and management interventions can be largely explained by differences in the relative contributions of mixed and preformed fatty acid synthesis [51,52,53,54,55,56,57,58,59,60,61,62,63,64,65,66,67]. Studies often report heterogeneous outcomes because dietary treatments do not affect all fatty acid pools uniformly. Fatty acids synthesized de novo and those produced via mixed pathways depend heavily on rumen fermentation and mammary gland activity, whereas preformed fatty acids are primarily influenced by dietary fat intake and body fat mobilization [10,51,52,53,54,55,56,57,58,59].

Dose–response patterns further reflect these distinct synthesis routes. Moderate increases in dietary fat may enhance milk fat yield by supplying preformed fatty acids without disrupting rumen fermentation [56]. However, higher inclusion rates, particularly of unsaturated fats, can suppress de novo and mixed fatty acid synthesis through altered rumen biohydrogenation, leading to nonlinear or negative responses in milk fat concentration [59]. As a result, similar dietary interventions may produce different outcomes depending on fat source, inclusion level, and basal diet composition [51,52,53,54,55,56,57,58,59,60,61,62,63,64,65,66,67].

Differences in population characteristics also contribute to inconsistent findings across studies [51,52,53,54,55,56,57,58,59,60]. Early lactation cows, which experience greater negative energy balance, rely more heavily on preformed fatty acids derived from adipose tissue mobilization, whereas mid- to late-lactation cows depend more on de novo and mixed synthesis supported by rumen-derived substrates [51,52]. Parity, production level, and health status further modify these relationships by influencing energy balance, rumen stability, and mammary synthetic capacity [59].

Integrating mixed and preformed fatty acid synthesis, therefore, provides a mechanistic framework to interpret variability in milk fat responses. This approach allows observed heterogeneity to be understood not as conflicting evidence, but as biologically meaningful differences arising from diet composition, dose, and cow physiological state [59].

However, the sustainability of supplementation strategies requires consideration of environmental impact, economic feasibility, and long-term animal health [109]. Future research should focus on optimizing rumen bypass technologies, integrating microbiome-targeted interventions, and developing high-omega-3 plant varieties to maximize milk enrichment efficiently [110].

Overall, dietary interventions to increase milk omega-3 content are effective, particularly when combining high-omega-3 sources with rumen protection and strategic feeding management [111]. The convergence of nutritional, microbiological, and technological approaches offers a practical pathway to produce functionally enriched milk that meets consumer demand while maintaining cow performance and health [112]. From a public-health standpoint, bovine milk is relatively inexpensive, stores and transports well, can be fortified (e.g., vitamin D), and fits into many cuisines, so governments and institutions could prefer it as a population-level supplement. Bovine milk is nutrient-dense, highly bioavailable, protein-rich, and easy to use at scale, and that makes it a very efficient default supplement [59]. Milk proteins score is very high on protein quality scales, contains all essential amino acids, whey is fast-digesting (great for muscle repair), casein digests slowly (supports sustained amino acid release), so milk and whey dominate sports nutrition and medical supplementation [53,54,55,56,57].

Based on current literature, several promising avenues are emerging due to the following reasons: (1) enhanced rumen protection technologies: improved microencapsulation, pH-responsive coatings, and other delivery systems to maximize transfer of ALA, EPA, and DHA [113], (2) microbiome-targeted strategies: further research into rumen microbial manipulation (e.g., with additives like tannins, essential oils) to limit bio hydrogenation bacteria, (3) genetically enriched oilseeds: development of oilseed crops with higher ALA or modified lipid profiles to deliver more omega-3 without compromising digestibility, (4) longer-term studies: many experiments are relatively short; more longitudinal studies are needed to assess animal health, reproduction, milk stability (oxidation), and consumer acceptability, (5) sustainability assessments: given the environmental footprint of marine-derived supplements, evaluating life-cycle sustainability of microalgae versus fish oil is important [114].

Limitations and gaps are that most trials report modest effect sizes, and heterogeneity in study design, dose, duration, and participant health status complicates firm conclusions. Many findings are observational (cannot prove causality), RCTs often show only small effects, and heterogeneity is common [115]. Benefits may be greater in older or at-risk populations (frailty, cognitive impairment) than in healthy, younger older adults. Some studies use blood levels/dietary intake; others use supplementation—these may differ in effect [74]. Mechanistic pathways (anti-inflammation, improved cell membrane fluidity, neuroprotection, muscle protein synthesis) are plausible but not fully clarified. The benefits are associative rather than definitive, and further large-scale, long-term RCTs are needed to determine optimal dosing and the populations most likely to benefit. Future research should also clarify the most beneficial dose for overall health and well-being in older adults. Despite advances in characterizing milk fatty acid synthesis, important knowledge gaps remain that limit the interpretation and application of existing findings. Many studies rely on aggregate milk fat measures, providing limited resolution on how mixed and preformed fatty acid pools respond independently to dietary interventions, particularly across different inclusion rates and fat sources. In addition, inconsistent reporting of cow physiological status (e.g., stage of lactation, parity, and energy balance) constrains comparisons among studies and likely contributes to observed heterogeneity in outcomes. Future research should prioritize dose–response designs that explicitly quantify changes in de novo, mixed, and preformed fatty acid synthesis, while integrating markers of rumen function and metabolic status. Longitudinal studies spanning the transition period and mid-lactation would further clarify how shifts in fatty acid origin mediate milk fat responses over time. Addressing these gaps will improve mechanistic understanding and support the development of more precise, physiology-based nutritional and management strategies.

## 5. Conclusions

The collective evidence from randomized controlled trials suggests that omega-3 polyunsaturated fatty acids (EPA and DHA) contribute modestly to healthy ageing, primarily through benefits to cardiovascular, cognitive, and musculoskeletal health. Regarding cognitive ageing, higher intakes of omega-3s—particularly at doses around 1–3 g/day of combined EPA and DHA over extended periods (≥12 months)—are associated with improvements in cognitive domains such as executive function, memory, and processing speed, especially among older adults with low baseline omega-3 status or existing cardiovascular disease. However, results across trials remain inconsistent, with smaller or shorter studies (<1 g/day, <6 months) often showing no significant effect. Omega-3 supplementation may support muscle health and physical performance by improving muscle protein synthesis and reducing inflammation, but measurable gains in muscle mass and functional mobility (e.g., faster timed up and go test results) at doses of ≥2 g/day of EPA and DHA, particularly over longer durations. Nonetheless, the effects on muscle strength alone are limited, and benefits are more apparent when combined with resistance training and adequate protein intake. Regular intake of omega-3 fatty acids—through diet or supplementation—appears to be a safe and potentially valuable strategy to promote healthy ageing by protecting cardiovascular and cognitive function and maintaining physical capacity. While omega-3s are not a stand-alone intervention, integrating them into a broader lifestyle pattern that includes a balanced diet, physical activity, and disease prevention may enhance both lifespan and health span in older adults.

Dietary interventions in dairy cows—including flaxseed (whole or ground), protected marine oils (e.g., tuna oil), and microalgae (e.g., *Schizochytrium*)—can substantially enhance omega-3 PUFA content (ALA, EPA, DHA) in milk. The most effective strategies combine rich omega-3 sources with rumen-protection technologies to overcome biohydrogenation. Such interventions not only improve the nutritional value of milk for human consumers but can also positively influence cow health, metabolism, and reproductive function. Continued innovation in protection methods, microbial modulation, and cost-effective supplementation will be key to scaling up omega-3–enriched milk production.

## Figures and Tables

**Table 1:** Details regarding the search process and the unique contributions of each database to this study.

Database	Keywords	MeSH Terms (PubMed)	Initial Articles	Duplicates Removed	Final Articles for Analysis	Contribution to Study	Reason for Inclusion
PubMed	#Health, #Diet, #Omega-3 polyunsaturated fatty acids, #Intervention, #Randomized clinical trial, #Older adults, #Ageing, #Cognitive function, #Muscle mass, #Physical function, #Strength, #Inflammation. #Dairy Cow, #Nutritional profile, and #Milk	#Omega-3 polyunsaturated fatty acids, #Intervention, #Randomized clinical trial, #Older adults, #Dairy Cow, #Nutritional profile, and #Milk	190	48	142	Provided a broad understanding of the interplay between diet, food consumption, dietary interventions, and mental health benefits; MeSH terms ensured precision in the search for relevant literature	Widely recognized as a premier biomedical database, frequently used for reviews in healthcare research
Web of Science	# Health, #Diet, #Omega-3 polyunsaturated fatty acids, #Intervention, #Randomized clinical trial, #Older adults, #Ageing, #Cognitive function, #Muscle Mass, #Physical Function, #Strength, and #Inflammation, #Mental health, #Exercise, #Lifestyle, #Cardiovascular disease, #Cancer, #Food, #Nutrition, #Dairy Cow, #Nutritional profile, and #Milk	N/A (Web of Science does not use MeSH terms)	5	3	2	Enhanced the overall coverage of literature related to dietary interventions and their impact on mental health	Provides a multidisciplinary approach, covering a wide range of scientific disciplines
Scopus	# Health, #Diet, #Omega-3 polyunsaturated fatty acids, #Intervention, #Randomized clinical trial, #Older adults, #Ageing, #Cognitive function, #Muscle mass, #Physical function, #Strength, and #Inflammation.	#Omega-3 polyunsaturated fatty acids, #Randomized clinical trial, #Health	12	9	3	Strengthened the evidence base by focusing on diet interventions and their impact on mental health; MeSH terms ensured specificity in selecting relevant literature	Renowned for reviews and emphasizing evidence-based interventions in healthcare research
Cochrane Library	#Omega-3 polyunsaturated fatty acids, #Interventions, #Nutrition, #Overall health, and #Randomized clinical trials, #Supplementation	#Omega-3 polyunsaturated fatty acids, #Interventions, #Health	30	28	2	Strengthened the evidence base by focusing on bioactive compounds in meals and snacks related to evidence-based interventions; MeSH terms ensured specificity in selecting relevant literature	Renowned for reviews and emphasizing evidence-based interventions in healthcare research

**Table 2:** Inclusion and exclusion criteria.

Inclusion Criteria	Exclusion Criteria
Published in English	Case reports and practical guidelines
Randomized controlled trials or reviews	Sample parameters (small sample)
Participants aged >60 years old	No comparator group (i.e., control or alternative dietary intervention)
Studies with a minimum of 3 months follow-up and a minimum of 24 participants	Studies published before 2010
In vitro and in vivo studies	Does not report primary and/or secondary outcomes

**Table 3:** Randomized clinical trials of long-chain omega-3 PUFA in older adults (population, dose, duration, outcome, effect size/remarks).

Authors and Aim	Population and Study Design	Dose	Duration	Outcome(s)	Effect Size/ Remarks	Reference
**Cognitive function**						
Power et al., 2022	RCT ^1^ in cognitively healthy individuals aged ≥65 years, *n* = 60	Daily 1 g fish oil (of which 430 mg DHA, 90 mg EPA), 22 mg carotenoids (10 mg lutein, 10 mg meso-zeaxanthin, 2 mg zeaxanthin), and 15 mg vitamin E.	24 months	Nutrients work synergistically, and in a dose-dependent manner, to improve working memory in cognitively healthy older adults.	Increasing nutritional intake of carotenoids and omega-3 fatty acids may prove beneficial in reducing cognitive decline and dementia risk in later life.	[22]
Danthiir et al., 2018	RCT ^1^ in cognitively healthy individuals aged ≥65 years, *n* = 60	1720 mg DHA and 600 mg eicosapentaenoic acid.	18 months	Treatment interactions with sex and APOE-ε4 carrier status warrant further investigation.	Daily supplementation with 2.3 g DHA-rich fish oil did not maintain or improve cognitive performance.	[23]
Malik et al., 2021	Older adults with stable coronary artery disease, cognitively healthy, *n* = 285	3.36 g/day (EPA + DHA)	30 months	Cognitive domains: verbal fluency, language, memory, and visual-motor coordination.	Significant improvement vs. control (mean ~1.08 pts; 95% CI 0.25–1.91).	[24]
Mahmoudi et al., 2014	Older people, normal or mildly impaired cognition, *n* = 199	Low dose: ~400 mg/day or ~600 mg DHA and EPA	26 weeks (~6 months)	Cognitive status (MMSE, etc.)	No overall therapeutic effect of low dose; benefits are not clinically meaningful	[68]
Geleijnse et al., 2012	Older adults with stable coronary artery disease, RCT ^1^; *n* = 2911, aged 60 to 80 years	400 mg/day EPA and DHA, 2 g/d of α-linolenic acid	40 months	Changes in Mini-Mental State Examination score during intervention did not differ significantly.	No effect of dietary doses of omega-3 fatty acids on global cognitive decline in coronary heart disease patients.	[26]
Kesse-Guyot et al., 2012	Older adults with prior cardiovascular disease, RCT ^1^; SU.FOL.OM3 trial, *n* = 2501, mean age 65.5 years	600 mg/day EPA and DHA	5 years	No significant improvement in global cognition or memory.	Low-to-moderate doses (<1 g/day) did not yield measurable cognitive benefits.	[27]
Dangour et al., 2010	Healthy older adults (≥70 years), OPAL Study, *n* = 867	700 mg/day EPA and DHA	24 months	No difference in cognitive decline vs. placebo.	Shorter or lower-dose interventions were largely ineffective.	[28]
Yurko-Mauro et al., 2010	Older adults with mild memory complaints, RCT ^1^, *n* = 485	900 mg/day DHA	24 weeks	Significant improvement in episodic memory and learning; no global cognition change.	Targeted cognitive domains may respond even at ~1 g/day DHA.	[29]
Sinn et al., 2011	RCT ^1^ in older adults with mild cognitive impairment, *n* = 50 people aged >65 years	A supplement rich in EPA (1.67 g EPA + 0.16 g DHA/d), DHA (1.55 g DHA + 0.40 g EPA/d), or the n-6 PUFA linoleic acid (2.2 g/d).	6 months	Increased intakes of DHA and EPA benefited mental health in older people with mild cognitive impairment.	Increasing omega-3 PUFA intakes may reduce depressive symptoms and the risk of progressing to dementia.	[30]
McNamara et al., 2018	*N* = 94, RCT ^1^ in older adult women (62–80 years)	Daily fish oil (four capsules, each of which contained 400 mg EPA and 200 mg DHA for total daily doses of 1.6 g EPA and 0.8 g DHA) or blueberry or both.	24 weeks	The fish oil (*p* = 0.03) and blueberry (*p* = 0.05) groups reported fewer cognitive symptoms, and the last group showed improved memory discrimination.	Combined treatment was not associated with cognitive enhancement as expected.	[31]
**Muscle Mass and Physical Function**						
Bischoff-Ferrari et al., 2020	*N* = 2157 adults without major comorbidities, mean age 74.9 years, The DO-HEALTH a	1 g/d of omega-3s,	2.99 years	The differences in mean change in systolic Blood pressure with omega-3s vs. no omega-3s were both −0.8 (99% CI, −2.1 to 0.5) mm Hg, with *p* < 0.13 and *p* < 0.11, respectively; the difference in mean change in diastolic blood pressure with omega-3s vs. no omega-3s was −0.5 (99% CI, −1.2 to 0.2) mm Hg; *p * = 0.06); and the difference in mean change in incidence rates of infections with omega-3s vs. no omega-3s was −0.13 (99% CI, −0.23 to −0.03), with an incidence rates ratio of 0.89 (99% CI, 0.78–1.01; *p* = 0.02)	Treatment with vitamin D3, omega-3s, or a strength-training exercise programme did not result in statistically significant differences in improvement in systolic or diastolic blood pressure, no vertebral fractures, physical performance, infection rates, or cognitive function.	[32]
Rolland et al., 2019	Older adults ≥ 70 years, non-demented, *n* = 1680 (from the MAPT trial)	“Low dose” omega-3-PUFA (not very high)	3 years	Hand-grip strength (muscle strength)	No significant effect of omega-3 alone or with lifestyle intervention on muscle strength	[33]
Smith et al., 2015	Healthy older adults, RCT ^1^, *n* = 60; mean age ~71 years	4 g/day EPA + DHA	6 months	Increased thigh muscle volume (+3.5%), hand-grip strength (+4%), and protein synthesis rates.	High-dose, long-term supplementation improved muscle anabolic response.	[34]
Rodacki et al., 2012	RCT ^1^, *n* = 45 older women	2 g/day fish oil	90 days and strength training	Enhanced torque and functional performance compared to training alone.	Omega-3s may augment resistance training effects.	[35]
Tardivo et al., 2015	*N* = 87 Brazilian women with metabolic syndrome (age ≥ 45 years and with amenorrhea ≥ 12 months)	900 mg/day omega-3	6 months	No significant changes in body fat or muscle mass	In postmenopausal women with metabolic syndrome, dietary intervention plus supplementation of omega-3 resulted in a further decrease in triglycerides and blood pressure and also in an improvement in insulin resistance and inflammatory markers, important components of metabolic syndrome.	[36]
Krzyminska-Siemaszko et al., 2015	*N* = 53, RCT ^1^, community- dwelling elderly aged ≥60 y old, with decreased muscle mass or at risk of low muscle mass.	1.3 g omega-3 PUFA (660 mg EPA, 440 mg DHA +200 mg other omega-3 PUFA)	12 weeks	No statistically significant differences in the analyzed components of body composition, in muscle strength nor in physical performance (4- Metre Walking Test and Go test) in any group.	A 12-week supplementation of PUFA did not affect the evaluated parameters in elderly individuals with decreased muscle mass.	[37]
Logan and Spriet, 2015	RCT ^1^, *n* = 24 healthy, community-dwelling older women aged 60–76 years old.	3 g omega-3 PUFA (2 g EPA, 1 g DHA)	12 weeks	Significantly increased lean body mass and physical function (decreasing Timed Get Up and Go Test).	Significantly increased resting metabolic rate by 14%, energy expenditure during exercise by 10%, and the rate of fat oxidation during rest by 19% and during exercise by 27%, lowered triglyceride levels by 29%, and increased lean mass by 4% and functional capacity by 7%.	[38]
Edholm et al., 2020	RCT ^1^, *n* = 63, mean age 67.5 ± 0.4 years old	The dietary plan was based on an intake of 44% carbohydrates (fibre intake > 25 g/day), 36% fat (mainly monounsaturated and polyunsaturated fatty acids), and 20% protein, with the following major adjustment: the omeg-6/omega-3 ratio < 2.	24 weeks	The explosive capacity in dynamic movements also increased, as evidenced by the significant changes in knee extension peak power.	There was a greater (*p* < 0.05) increase in muscle quality in women after exercise training in the long-chain n–3 PUFA group than in the placebo group, with no such differences in men.	[39]
**Overall health**						
Manson et al., 2019	Middle-aged/older adults (mean age ~67 years), *n* = 25,871 (VITAL Trial), men 50 years of age or older, and women 55 years of age or older	1 g/day EPA + DHA	5 years	Reduced risk of major cardiac events (in subgroups with low fish intake); no cognitive or physical function benefit.	Supports cardiovascular protection, not necessarily direct anti-ageing benefits.	[40]
Fakhrzadeh et al., 2010	*N* = 124, RCT ^1^, elderly ≥ 65 years old	1 g/day fish oil capsule (with 180 mg eicosapentaenoic acid, EPA; and 120 mg docosahexaenoic acid, DHA; a total of 300 mg omega-3 fatty acids as effective constituents.	6 months	The overall decrease in serum triglycerides compared with placebo was significant (*p* = 0.04).	Supplementation with low-dose omega-3 fatty acids for 6 months could significantly protect elderly Iranians from a rise in serum triglycerides.	[41]
Ballantyne et al., 2012	RCT ^1^ in statin-treated patients with persistent high triglycerides, *n* = 702, (ANCHOR study), mean age 61.5 years	4 and 2 g/day AMR101	12 weeks	AMR101 4 g/day decreased LDL ** cholesterol by 6.2% (*p* = 0.0067) and decreased apo lipoprotein B (9.3%), total cholesterol (12.0%), very-low-density lipoprotein cholesterol (24.4%), lipoprotein-associated phospholipase A(2) (19.0%), and high-sensitivity C-reactive protein (22.0%) versus placebo (*p* < 0.001 for all comparisons).	Significantly decreased median placebo-adjusted TG ***, non-HDL cholesterol, LDL cholesterol, apo lipoprotein B, total cholesterol, very-low-density lipoprotein cholesterol, lipoprotein-associated phospholipase A(2), and high-sensitivity C-reactive protein in statin-treated patients with residual TG elevations.	[42]
Deepak et al., 2018	*N* = 8179 patients with established cardiovascular disease or with diabetes and other risk factors (REDUCE-IT)	2 g of icosapent ethyl twice daily (total daily dose, 4 g)	4.9 years	The rates of additional ischemic end points, as assessed according to a prespecified hierarchical schema, were significantly lower in the icosapent ethyl group than in the placebo group, including the rate of cardiovascular death	The risk of ischemic events, including cardiovascular death, was significantly lower among those who received 2 g of icosapent ethyl twice daily	[43]
Alfaddagh et al., 2017	*N* = 850 patients with coronary artery disease, RCT ^1,^ mean age 63.3 years	1.86 g of EPA and 1.5 g of DHA daily	30 months	Among those on low-intensity statins, omega-3 ethyl-ester subjects had attenuation of fibrous plaque progression compared to controls (median% change [interquartile range], 0.3% [−12.8, 9.0] versus 4.8% [−5.1, 19.0], respectively; *p* = 0.032). In contrast, those on high-intensity statins had no difference in plaque change in either treatment arm.	The benefit on low-intensity statin, but not high-intensity statin, suggests that statin intensity affects plaque volume.	[44]
Nicholls et al., 2020	*N* = 13,078 patients, with high cardiovascular risk, high triglycerides, and low HDL * cholesterol levels, mean age 62.5 years (The STRENGTH RCT ^1^)	4 g/d of omega-3 fatty acids	12 months	The addition of omega-3 CA, compared with corn oil, to usual background therapies resulted in no significant difference in a composite outcome of major adverse cardiovascular events.	These findings do not support the use of this omega-3 fatty acid formulation to reduce major adverse cardiovascular events in patients with high cardiovascular risk.	[45]
Budoff et al., 2020	*N* = 80 patients with elevated triglycerides on statin therapy, mean age 58.3 years (EVAPORATE trial)	4 g/day icosapent ethyl	18 months	Reduce initial cardiovascular events by 25% and total cardiovascular events by 32%.	Great change, but the mechanisms of benefit are not yet fully explained.	[46]
Ando et al., 2015	*N* = 200 coronary heart disease patients, RCT ^1^, age > 60 years	High dose pitavastatin therapy, 4 mg/day and EPA 1800 mg/day	6–8 months	Plaque regression was defined as a percent change in plaque volume of more than −14.6% according to previous reports.	The prevalence rate of plaque regression was significantly higher in the PTV/EPA group than in the PTV group.	[47]
Costenbader et al., 2019	*N* = 1561, women ≥ 55 and men ≥ 50 years of age, RCT ^1^ (Vitamin D and Omega-3 Trial)	Vitamin D (2000 IU/day) and/or omega-3 fatty acids (1 g/day)	1 year	Among 777 randomized to omega-3 FA, hsCRP ^2^ declined [−10.5% (−20.4% to 0.8%)] in those with baseline low (<1.5 servings/week), but not with higher fish intake [6.4% (95% CI, −7.11% to 21.8%); *p* interaction = 0.06].	Neither vitamin D nor omega-3 FA supplementation over 1 year decreased these biomarkers of inflammation.	[48]
McDonald et al., 2014	*N* = 153, women who have completed breast cancer treatment, >56 years, RCT ^1^	3g/d long chain omega-3 fatty acids (1.75 g EPA and 1.25 g DHA)	12 months	Long-chain omega-3 fatty acids alone or in combination with exercise in breast cancer survivors with regard to lean body mass and quality of life.	Improve evidence-based dietetic practice.	[49]
Filip et al., 2015	*N* = 64 osteopenic patients RCT ^1^	250 mg/day of olive extract and 1000 mg Calcium	12 months	Significant decrease in total- and LDL **-cholesterol in the treatment group.	The improved blood lipid profiles suggest additional health benefits associated with the intake of the olive polyphenol extract	[50]

^1^ RCT: Randomized clinical trial. AMR101 is an omega-3 fatty acid agent containing ≥ 96% pure icosapent-ethyl, the ethyl ester of eicosapentaenoic acid, * HDL: high-density lipoprotein, ** LDL: low-density lipoprotein, *** TG: triglyceride, ^2^ hsCRP: high-sensitivity C-reactive protein.

**Table 4:** Expected changes in milk fatty acid composition with diet interventions: pasture, flaxseed, linseed oil, fish oil, rumen-protected fats [51,52,53,54,55,56,57,58,59,60,61,62,63,64,65,66,67].

Component	Baseline	Expected After Intervention	Change ^1^
α-Linolenic acid (ALA; C18:3 omega-3)	0.3–0.7% of total fatty acids	0.8–2.0%	More than 2–4%
EPA (C20:5 omega-3)	<0.05%	0.05–0.2% ^1^	More than 2–5%
DHA (C22:6 omega-3)	<0.02%	0.03–0.1%	More than 2–5%

^1^ Fish oil causes the largest jump in DHA/EPA; flaxseed mainly increases ALA.

**Table 5:** Expected changes in milk fatty acid composition with diet interventions: asture, sunflower oil/meal, linseed oil, and fish oil combinations [80].

Component	Baseline	Expected After Intervention	Change ^1^
cis-9, trans-11 CLA	0.3–0.6% of total fatty acids	0.7–1.3%	More than 30–100%
trans-11 vaccenic acid (C18:1 t11)	1–3%	2–6% ^1^	More than 50–150%

^1^ Vaccenic acid is a conjugated linoleic acid precursor; more vaccenic acid = more conjugated linoleic acid in milk.

**Table 6:** Expected changes in milk fatty acid composition with diet interventions: flaxseed, canola, high-quality pasture, protected oils [51,52,53,54,55,56,57,58,59,60,61,62,63,64,65,66,67].

Component	Baseline	Expected After Intervention	Change
Total unsaturated FA	28–33% of total fatty acids	32–40%	More than 10–20%
Mono-unsaturated fats (MUFA)	22–26%	24–30%	More than 5–15%
Polyunsaturated fats (PUFA)	3–4%	4–6%	More than 25–50%
Total saturated fatty acids	67–72%	60–80%	Lower than 5–12%
Palmitic acid (C16:0)	28–33%	24–30%	Lower than 5–15%

**Figure A1:**
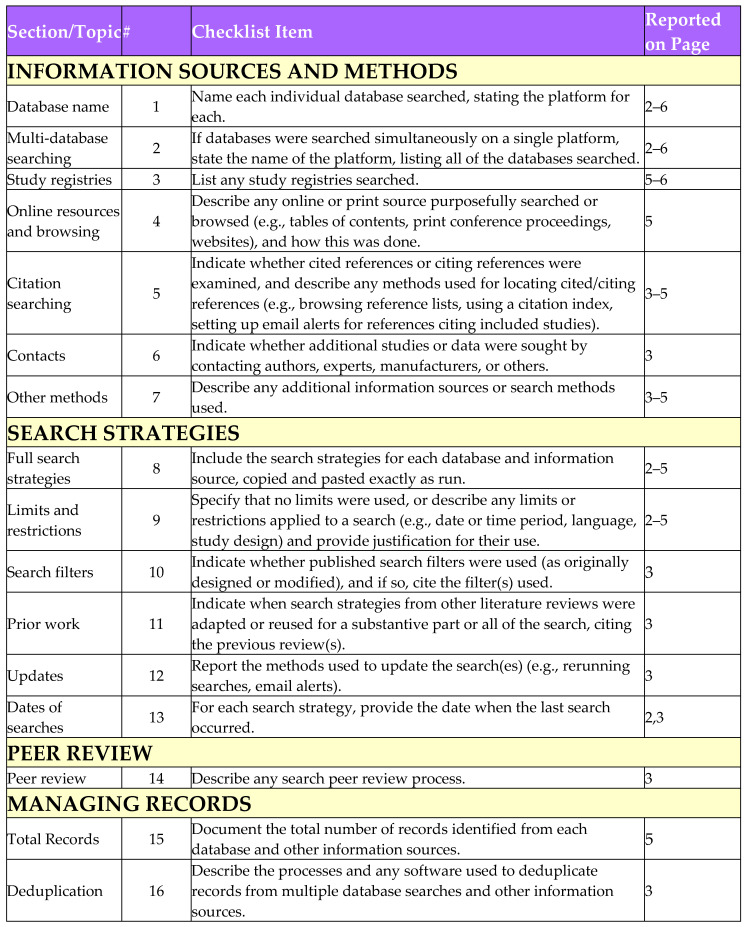
PRISMA Checklist [19].

## Data Availability

No new data were created or analyzed in this study. Data sharing is not applicable to this article.

## Supplementary Materials

The following supporting information can be downloaded at: https://www.mdpi.com/article/10.3390/foods15061079/s1, Table S1: PICOTS model (Population, Type of intervention, Comparator, Outcomes, Time factor, and Study design).

## References

[1] Officer A., Schneiders M.L., Wu D., Nash P., Thiyagarajan J.A., Beard J.R. (2016). Valuing older people: Time for a global campaign to combat ageism. Bull. World Health Organ..

[2] Noto S. (2023). Perspectives on Aging and Quality of Life. Healthcare.

[3] Preston J., Biddell B. (2021). The physiology of ageing and how these changes affect older people. Medicine.

[4] Bordoni L., Petracci I., Zhao F., Min W., Pierella E., Assmann T.S., Martinez J.A., Gabbianelli R. (2021). Nutrigenomics of dietary lipids. Antioxidants.

[5] Lagoumintzis G., Patrinos G.P. (2023). Triangulating nutrigenomics, metabolomics and microbiomics toward personalized nutrition and healthy living. Hum. Genom..

[6] Sung H., Vaziri A., Wilinski D., Woerner R.K., Freddolino L., Dus M. (2023). Nutrigenomic regulation of sensory plasticity. eLife.

[7] Donato K., Madeo G., Micheletti C., Cristoni S., Ceccarini M., Beccari T., Iaconelli A., Aquilanti B., Matera G., Herbst K. (2023). Nutrigenomics: SNPs correlated to physical activity, response to chiropractic treatment, mood and sleep. Clin. Ter..

[8] Erickson N., Sulosaari V., Sullivan E.S., Laviano A., van Ginkel-Res A., Remijnse W., Wesseling J., Koepcke U., Weber N., Huebner J. (2025). Nutrition Care in Cancer: An Overlooked Part of Patient-Centered Care. Semin. Oncol. Nurs..

[9] D’Eliseo D., Velotti F. (2016). Omega-3 fatty acids and cancer cell cytotoxicity: Implications for multi-targeted cancer therapy. J. Clin. Med..

[10] Berglin E., Dahlqvist S.R. (2013). Comparison of the 1987 ACR and 2010 ACR/EULAR classification criteria for rheumatoid arthritis in clinical practice: A prospective cohort study. Scand. J. Rheumatol..

[11] Mititelu M., Lupuliasa D., Neacșu S.M., Olteanu G., Busnatu Ș.S., Mihai A., Popovici V., Măru N., Boroghină S.C., Mihai S. (2024). Polyunsaturated Fatty Acids and Human Health: A Key to Modern Nutritional Balance in Association with Polyphenolic Compounds from Food Sources. Foods.

[12] DiNicolantonio J.J., O’Keefe J. (2021). The Importance of Maintaining a Low Omega-6/Omega-3 Ratio for Reducing the Risk of Autoimmune Diseases, Asthma, and Allergies. Mo. Med..

[13] Patted P.G., Masareddy R.S., Patil A.S., Kanabargi R.R., Bhat C.T. (2024). Omega-3 fatty acids: A comprehensive scientific review of their sources, functions and health benefits. Future J. Pharm. Sci..

[14] Mukherjee S., Mohanty A.K., Rao U., Poddar A. (2024). Microbial polyunsaturated fatty acids (PUFAs) for human health: A comprehensive review of evidence from in vivo preclinical models. Discov. Appl. Sci..

[15] Benbrook C.M., Butler G., Latif M.A., Leifert C., Davis D.R. (2013). Organic production enhances milk nutritional quality by shifting fatty acid composition: A United States–wide, 18-month study. PLoS ONE.

[16] Huang G., Li N., Wu X., Zheng N., Zhao S., Zhang Y., Wang J. (2024). Nutrition, production, and processing of virgin omega-3 polyunsaturated fatty acids in dairy: An integrative review. Heliyon.

[17] Caroprese M., Marzano A., Marino R., Gliatta G., Muscio A., Sevi A. (2010). Flaxseed supplementation improves fatty acid profile of cow milk. J. Dairy Sci..

[18] Radzikowska U., Rinaldi A.O., Çelebi Sözener Z., Karaguzel D., Wojcik M., Cypryk K., Akdis M., Akdis C.A., Sokolowska M. (2019). The influence of dietary fatty acids on immune responses. Nutrients.

[19] Page M.J., McKenzie J.E., Bossuyt P.M., Boutron I., Hoffmann T.C., Mulrow C.D., Shamseer L., Tetzlaff J.M., Akl E.A., Brennan S.E. (2021). The PRISMA 2020 statement: An updated guideline for reporting systematic reviews. BMJ.

[20] Grossman J., Mackenzie F.J. (2005). The randomized controlled trial: Gold standard, or merely standard?. Perspect. Biol. Med..

[21] Higgins J.P., Altman D.G., Gøtzsche P.C., Jüni P., Moher D., Oxman A.D., Savović J., Schulz K.F., Weeks L., Sterne J.A. (2011). The Cochrane Collaboration’s tool for assessing risk of bias in randomised trials. BMJ.

[22] Power R., Nolan J.M., Prado-Cabrero A., Roche W., Coen R., Power T., Mulcahy R. (2022). Omega-3 fatty acid, carotenoid and vitamin E supplementation improves working memory in older adults: A randomised clinical trial. Clin. Nutr..

[23] Danthiir V., Hosking D.E., Nettelbeck T., Vincent A.D., Wilson C., O’Callaghan N., Calvaresi E., Clifton P., Wittert G.A. (2018). An 18-mo randomized, double-blind, placebo-controlled trial of DHA-rich fish oil to prevent age-related cognitive decline in cognitively normal older adults. Am. J. Clin. Nutr..

[24] Malik A., Ramadan A., Vemuri B., Siddiq W., Amangurbanova M., Ali A., Welty F.K. (2021). ω-3 Ethyl ester results in better cognitive function at 12 and 30 months than control in cognitively healthy subjects with coronary artery disease: A secondary analysis of a randomized clinical trial. Am. J. Clin. Nutr..

[25] Mahmoudi M.J., Hedayat M., Sharifi F., Mirarefin M., Nazari N., Mehrdad N., Ghaderpanahi M., Tajalizadekhoob Y., Badamchizade Z., Larijani B. (2014). Effect of low dose ω-3 poly unsaturated fatty acids on cognitive status among older people: A double-blind randomized placebo-controlled study. J. Diabetes Metab. Disord..

[26] Geleijnse J.M., Giltay E.J., Kromhout D. (2012). Effects of n-3 fatty acids on cognitive decline: A randomized, double-blind, placebo-controlled trial in stable myocardial infarction patients. Alzheimers Dement..

[27] Kesse-Guyot E., Touvier M., Andreeva V.A., Jeandel C., Ferry M., Hercberg S., Galan P. (2012). Cross-sectional but not longitudinal association between n-3 fatty acid intake and depressive symptoms: Results from the SU. VI. MAX 2 study. Am. J. Epidemiol..

[28] Dangour A.D., Allen E., Elbourne D., Fasey N., Fletcher A.E., Hardy P., Holder G.E., Knight R., Letley L., Richards M. (2010). Effect of 2-y n-3 long-chain polyunsaturated fatty acid supplementation on cognitive function in older people: A randomized, double-blind, controlled trial. Am. J. Clin. Nutr..

[29] Yurko-Mauro K. (2010). Cognitive and cardiovascular benefits of docosahexaenoic acid in aging and cognitive decline. Curr. Alzheimer Res..

[30] Sinn N., Milte C.M., Street S.J., Buckley J.D., Coates A.M., Petkov J., Howe P.R.C. (2012). Effects of n-3 fatty acids, EPA v. DHA, on depressive symptoms, quality of life, memory and executive function in older adults with mild cognitive impairment: A 6-month randomised controlled trial. Br. J. Nutr..

[31] McNamara R.K., Kalt W., Shidler M.D., McDonald J., Summer S.S., Stein A.L., Stover A.N., Krikorian R. (2018). Cognitive response to fish oil, blueberry, and combined supplementation in older adults with subjective cognitive impairment. Neurobiol. Aging.

[32] Bischoff-Ferrari H.A., Vellas B., Rizzoli R., Kressig R.W., da Silva J.A.P., Blauth M., Felson D.T., McCloskey E.V., Watzl B., Hofbauer L.C. (2020). Effect of Vitamin D Supplementation, Omega-3 Fatty Acid Supplementation, or a Strength-Training Exercise Program on Clinical Outcomes in Older Adults: The DO-HEALTH Randomized Clinical Trial. JAMA.

[33] Rolland Y., Barreto P.d.S., Maltais M., Guyonnet S., Cantet C., Andrieu S., Vellas B. (2019). Effect of long-term omega 3 polyunsaturated fatty acid supplementation with or without multidomain lifestyle intervention on muscle strength in older adults: Secondary analysis of the Multidomain Alzheimer Preventive Trial (MAPT). Nutrients.

[34] Smith G.I., Julliand S., Reeds D.N., Sinacore D.R., Klein S., Mittendorfer B. (2015). Fish oil–derived n−3 PUFA therapy increases muscle mass and function in healthy older adults. Am. J. Clin. Nutr..

[35] Rodacki C.L., Rodacki A.L., Pereira G., Naliwaiko K., Coelho I., Pequito D., Fernandes L.C. (2012). Fish-oil supplementation enhances the effects of strength training in elderly women. Am. J. Clin. Nutr..

[36] Tardivo A.P., Nahas-Neto J., Orsatti C.L., Dias F.B., Poloni P.F., Schmitt E.B., Nahas E.A.P. (2015). Effects of omega-3 on metabolic markers in postmenopausal women with metabolic syndrome. Climacteric.

[37] Krzymińska-Siemaszko R., Czepulis N., Lewandowicz M., Zasadzka E., Suwalska A., Witowski J., Wieczorowska-Tobis K. (2015). The Effect of a 12-Week Omega-3 Supplementation on Body Composition, Muscle Strength and Physical Performance in Elderly Individuals with Decreased Muscle Mass. Int. J. Environ. Res. Public Health.

[38] Logan S.L., Spriet L.L. (2015). Omega-3 fatty acid supplementation for 12 weeks increases resting and exercise metabolic rate in healthy community-dwelling older females. PLoS ONE.

[39] Edholm P., Strandberg E., Kadi F. (2017). Lower limb explosive strength capacity in elderly women: Effects of resistance training and healthy diet. J. Appl. Physiol..

[40] Manson J.E., Cook N.R., Lee I.-M., Christen W., Bassuk S.S., Mora S., Gibson H., Albert C.M., Gordon D., Copeland T. (2019). Marine n− 3 fatty acids and prevention of cardiovascular disease and cancer. N. Engl. J. Med..

[41] Fakhrzadeh H., Ghaderpanahi M., Sharifi F., Mirarefin M., Badamchizade Z., Kamrani A.A., Larijani B. (2010). The effects of low dose n-3 fatty acids on serum lipid profiles and insulin resistance of the elderly: A randomized controlled clinical trial. Int. J. Vitam. Nutr. Res..

[42] Ballantyne C.M., Bays H.E., Kastelein J.J., Stein E., Isaacsohn J.L., Braeckman R.A., Soni P.N. (2012). Efficacy and safety of eicosapentaenoic acid ethyl ester (AMR101) therapy in statin-treated patients with persistent high triglycerides (from the ANCHOR study). Am. J. Cardiol..

[43] Bhatt D.L., Steg P.G., Miller M., Brinton E.A., Jacobson T.A., Ketchum S.B., Doyle R.T., Juliano R.A., Jiao L., Granowitz C. (2019). Cardiovascular Risk Reduction with Icosapent Ethyl for Hypertriglyceridemia. N. Engl. J. Med..

[44] Alfaddagh A., Elajami T.K., Ashfaque H., Saleh M., Bistrian B.R., Welty F.K. (2017). Effect of Eicosapentaenoic and Docosahexaenoic Acids Added to Statin Therapy on Coronary Artery Plaque in Patients with Coronary Artery Disease: A Randomized Clinical Trial. J. Am. Heart Assoc..

[45] Nicholls S.J., Lincoff A.M., Garcia M., Bash D., Ballantyne C.M., Barter P.J., Davidson M.H., Kastelein J.J.P., Koenig W., McGuire D.K. (2020). Effect of High-Dose Omega-3 Fatty Acids vs. Corn Oil on Major Adverse Cardiovascular Events in Patients at High Cardiovascular Risk: The STRENGTH Randomized Clinical Trial. JAMA.

[46] Budoff M.J., Bhatt D.L., Kinninger A., Lakshmanan S., Muhlestein J.B., Le V.T., May H.T., Shaikh K., Shekar C., Roy S.K. (2020). Effect of icosapent ethyl on progression of coronary atherosclerosis in patients with elevated triglycerides on statin therapy: Final results of the EVAPORATE trial. Eur. Heart J..

[47] Ando K., Watanabe T., Daidoji H., Otaki Y., Hashimoto N., Kumagai Y., Hashimoto N., Narumi T., Kadowaki S., Yamaura G. (2015). Abstract 12007: Combination Therapy of Eicosapentaenoic Acid and Pitavastatin for Coronary Plaque Regression Evaluated by Integrated Backscatter Intravascular Ultrasonography: A Randomized Controlled Trial. Circulation.

[48] Costenbader K.H., MacFarlane L.A., Lee I.-M., Buring J.E., Mora S., Bubes V., Kotler G., Camargo C.A., Manson J.E., Cook N.R. (2019). Effects of One Year of Vitamin D and Marine Omega-3 Fatty Acid Supplementation on Biomarkers of Systemic Inflammation in Older US Adults. Clin. Chem..

[49] McDonald C., Bauer J., Capra S., Coll J. (2014). The muscle mass, omega-3, diet, exercise and lifestyle (MODEL) study—A randomised controlled trial for women who have completed breast cancer treatment. BMC Cancer.

[50] Filip R., Possemiers S., Heyerick A., Pinheiro I., Raszewski G., Davicco M.J., Coxam V. (2015). Twelve-month consumption of a polyphenol extract from olive (*Olea europaea*) in a double blind, randomized trial increases serum total osteocalcin levels and improves serum lipid profiles in postmenopausal women with osteopenia. J. Nutr. Health Aging.

[51] Saadaoui I., Rasheed R., Aguilar A., Cherif M., Al Jabri H., Sayadi S., Manning S.R. (2021). Microalgal-based feed: Promising alternative feedstocks for livestock and poultry production. J. Anim. Sci. Biotechnol..

[52] Wathes D.C., Cheng Z.R., Marei W., Fouladi-Nashta A. (2013). Polyunsaturated fatty acids and fertility in female mammals: An update. CABI Rev..

[53] Șanta A., Mierlita D., Dărăban S., Socol C.T., Vicas S.I., Șuteu M., Maerescu C.M., Stanciu A.S., Pop I.M. (2022). The effect of sustainable feeding systems, combining total mixed rations and pasture, on milk fatty acid composition and antioxidant capacity in jersey dairy cows. Animals.

[54] Koçoğlu Y., Köknaroğlu H., Yalçın S. (2025). Effects of using different levels of flaxseed in dairy cattle diet on production performance and milk fatty acid profile. Trop. Anim. Health Prod..

[55] Huang G., Wang J., Liu K., Wang F., Zheng N., Zhao S., Qu X., Yu J., Zhang Y., Wang J. (2022). Effect of Flaxseed Supplementation on Milk and Plasma Fatty Acid Composition and Plasma Parameters of Holstein Dairy Cows. Animals.

[56] Huang G., Guo L., Chang X., Liu K., Tang W., Zheng N., Zhao S., Zhang Y., Wang J. (2021). Effect of Whole or Ground Flaxseed Supplementation on Fatty Acid Profile, Fermentation, and Bacterial Composition in Rumen of Dairy Cows. Front. Microbiol..

[57] Zhang H., He B., Luo J.Z., Liu Z.Y., Duan H.W., Ye H.B., Zhang J.Y., Ren D.X., Erdene K., Zhu W.Y. (2025). Supplementation of rumen-protected microalgae high in docosahexaenoic acid to lactating dairy cows: Dynamics of lactation performance, milk and plasma fatty acids, and transfer rate of docosahexaenoic acid to milk. J. Dairy Sci..

[58] Kokić B., Rakita S., Vujetić J. (2024). Impact of Using Oilseed Industry Byproducts Rich in Linoleic and Alpha-Linolenic Acid in Ruminant Nutrition on Milk Production and Milk Fatty Acid Profile. Animals.

[59] Sato Y., Tominaga K., Aoki H., Murayama M., Oishi K., Hirooka H., Yoshida T., Kumagai H. (2020). Calcium salts of long-chain fatty acids from linseed oil decrease methane production by altering the rumen microbiome in vitro. PLoS ONE.

[60] Liu X., Zhang X., He Q., Sun X., Wang W., Li S. (2025). Effects of increasing n3:n6 ratio by replacing extruded soybeans with extruded flaxseed on dry matter intake, rumen fluid bacteria, and liver lipid metabolism in transition cows. BMC Microbiol..

[61] Isenberg B., Soder K., Pereira A., Standish R., Brito A. (2019). Production, milk fatty acid profile, and nutrient utilization in grazing dairy cows supplemented with ground flaxseed. J. Dairy Sci..

[62] Fabjanowska J., Kowalczuk-Vasilev E., Klebaniuk R., Milewski S., Gümüş H. (2023). N-3 Polyunsaturated Fatty Acids as a Nutritional Support of the Reproductive and Immune System of Cattle—A Review. Animals.

[63] Beck M.R. (2020). Dietary Phytochemical Diversity to Enhance Health, Welfare and Production of Grazing Ruminants, While Reducing Environmental Impact. Ph.D. Thesis.

[64] Suksombat W., Meeprom C., Mirattanaphrai R. (2013). Milk production, milk composition, live weight change and milk Fatty Acid composition in lactating dairy cows in response to whole linseed supplementation. Asian-Australas. J. Anim. Sci..

[65] Villeneuve M.-P., Lebeuf Y., Gervais R., Tremblay G., Vuillemard J., Fortin J., Chouinard P. (2013). Milk volatile organic compounds and fatty acid profile in cows fed timothy as hay, pasture, or silage. J. Dairy Sci..

[66] Sterk A., Johansson B.E.O., Taweel H.Z.H., Murphy M., van Vuuren A.M., Hendriks W.H., Dijkstra J. (2011). Effects of forage type, forage to concentrate ratio, and crushed linseed supplementation on milk fatty acid profile in lactating dairy cows. J. Dairy Sci..

[67] Stepanchenko N., Stefenoni H., Hennessy M., Nagaraju I., Wasson D.E., Cueva S.F., Räisänen S.E., Dechow C.D., Pitta D.W., Hristov A.N. (2023). Microbial composition, rumen fermentation parameters, enteric methane emissions, and lactational performance of phenotypically high and low methane-emitting dairy cows. J. Dairy Sci..

[68] Tokuda H., Sueyasu T., Kontani M., Kawashima H., Shibata H., Koga Y. (2015). Low doses of long-chain polyunsaturated fatty acids affect cognitive function in elderly Japanese men: A randomized controlled trial. J. Oleo Sci..

[69] DiNicolantonio J.J., O’Keefe J.H. (2019). The Benefits of Marine Omega-3s for the Prevention and Treatment of Cardiovascular Disease. Mo. Med..

[70] Suh S.W., Lim E., Burm S.-Y., Lee H., Bae J.B., Han J.W., Kim K.W. (2024). The influence of n-3 polyunsaturated fatty acids on cognitive function in individuals without dementia: A systematic review and dose–response meta-analysis. BMC Med..

[71] Rondanelli M., Perna S., Riva A., Petrangolini G., Di Paolo E., Gasparri C. (2021). Effects of n-3 EPA and DHA supplementation on fat free mass and physical performance in elderly. A systematic review and meta-analysis of randomized clinical trial. Mech. Ageing Dev..

[72] Grenon S.M., Owens C.D., Nosova E.V., Hughes-Fulford M., Alley H.F., Chong K., Perez S., Yen P.K., Boscardin J., Hellmann J. (2015). Short-Term, High-Dose Fish Oil Supplementation Increases the Production of Omega-3 Fatty Acid-Derived Mediators in Patients with Peripheral Artery Disease (the OMEGA-PAD I Trial). J. Am. Heart Assoc..

[73] Da Boit M., Sibson R., Sivasubramaniam S., Meakin J.R., Greig C.A., Aspden R.M., Thies F., Jeromson S., Hamilton D.L., Speakman J.R. (2017). Sex differences in the effect of fish-oil supplementation on the adaptive response to resistance exercise training in older people: A randomized controlled trial. Am. J. Clin. Nutr..

[74] Strike S., Carlisle A., Gibson E., Dyall S. (2015). A High Omega-3 Fatty Acid Multinutrient Supplement Benefits Cognition and Mobility in Older Women: A Randomized, Double-blind, Placebo-controlled Pilot Study. J. Gerontol..

[75] Siddiqui R.A., Shaikh S.R., Sech L.A., Yount H.R., Stillwell W., Zaloga G.P. (2004). Omega 3-fatty acids: Health benefits and cellular mechanisms of action. Mini Rev. Med. Chem..

[76] Frumuzachi O., Gavrilaș L.I., Vodnar D.C., Rohn S., Mocan A. (2024). Systemic Health Effects of Oleuropein and Hydroxytyrosol Supplementation: A Systematic Review of Randomized Controlled Trials. Antioxidants.

[77] Bays H.E., Tighe A.P., Sadovsky R., Davidson M.H. (2008). Prescription omega-3 fatty acids and their lipid effects: Physiologic mechanisms of action and clinical implications. Expert Rev. Cardiovasc. Ther..

[78] Shibabaw T. (2021). Omega-3 polyunsaturated fatty acids: Anti-inflammatory and anti-hypertriglyceridemia mechanisms in cardiovascular disease. Mol. Cell. Biochem..

[79] Kitessa S.M., Gulati S.K., Simos G.C., Ashes J.R., Scott T.W., Fleck E., Wynn P.C. (2004). Supplementation of grazing dairy cows with rumen-protected tuna oil enriches milk fat with n-3 fatty acids without affecting milk production or sensory characteristics. Br. J. Nutr..

[80] Singh D., Amarjeet, Jha B., Pathak A., Rana T. (2025). Chapter 11—Role of fatty acids in milk production. Handbook of Milk Production, Quality and Nutrition.

[81] Giacobbe J., Benoiton B., Zunszain P., Pariante C.M., Borsini A. (2020). The anti-inflammatory role of omega-3 polyunsaturated fatty acids metabolites in pre-clinical models of psychiatric, neurodegenerative, and neurological disorders. Front. Psychiatry.

[82] Lipkovich I., Svensson D., Ratitch B., Dmitrienko A. (2024). Modern approaches for evaluating treatment effect heterogeneity from clinical trials and observational data. Stat. Med..

[83] Tanaka K., Farooqui A.A., Siddiqi N.J., Alhomida A.S., Ong W.Y. (2012). Effects of docosahexaenoic Acid on neurotransmission. Biomol. Ther..

[84] Shahinfar H., Yazdian Z., Avini N.A., Torabinasab K., Shab-Bidar S. (2025). A systematic review and dose response meta analysis of Omega 3 supplementation on cognitive function. Sci. Rep..

[85] Zhang X.W., Hou W.S., Li M., Tang Z.Y. (2016). Omega-3 fatty acids and risk of cognitive decline in the elderly: A meta-analysis of randomized controlled trials. Aging Clin. Exp. Res..

[86] Brainard J.S., Jimoh O.F., Deane K.H.O., Biswas P., Donaldson D., Maas K., Abdelhamid A.S., Hooper L. (2020). Omega-3, Omega-6, and Polyunsaturated Fat for Cognition: Systematic Review and Meta-analysis of Randomized Trials. J. Am. Med. Dir. Assoc..

[87] Alex A., Abbott K.A., McEvoy M., Schofield P.W., Garg M.L. (2020). Long-chain omega-3 polyunsaturated fatty acids and cognitive decline in non-demented adults: A systematic review and meta-analysis. Nutr. Rev..

[88] Nilsson A., Radeborg K., Salo I., Björck I. (2012). Effects of supplementation with n-3 polyunsaturated fatty acids on cognitive performance and cardiometabolic risk markers in healthy 51 to 72 years old subjects: A randomized controlled cross-over study. Nutr. J..

[89] Jordan P.M., Peltner L.K., Bachmann V., Dahlke P., Pace S., Thost L., Hörcher L.C., Nischang V., Temml V., Rossi A. (2025). Uncovering anti-inflammatory natural products that synergize with supplemented omega-3 PUFA for eliciting endogenous inflammation resolution signals. Biomed. Pharmacother..

[90] Lai H.T., de Oliveira Otto M.C., Lemaitre R.N., McKnight B., Song X., King I.B., Chaves P.H., Odden M.C., Newman A.B., Siscovick D.S. (2018). Serial circulating omega 3 polyunsaturated fatty acids and healthy ageing among older adults in the Cardiovascular Health Study: Prospective cohort study. BMJ.

[91] EFSA Panel on Dietetic Products, Nutrition and Allergies (NDA) (2016). DHA and improvement of memory function: Evaluation of a health claim pursuant to Article 13 (5) of Regulation (EC) No 1924/2006. EFSA J..

[92] Huang Y.H., Chiu W.C., Hsu Y.P., Lo Y.L., Wang Y.H. (2020). Effects of Omega-3 Fatty Acids on Muscle Mass, Muscle Strength and Muscle Performance among the Elderly: A Meta-Analysis. Nutrients.

[93] Santo André H.C., Esteves G.P., Barreto G.H.C., Longhini F., Dolan E., Benatti F.B. (2023). The Influence of n-3PUFA Supplementation on Muscle Strength, Mass, and Function: A Systematic Review and Meta-Analysis. Adv. Nutr..

[94] Dam D.L., Christensen J.A., Olsen P.Ø., Wilson J.J., Tully M.A., Buhl S.F., Caserotti P. (2025). Impact of Omega-3 Fatty Acids Supplementation Combined with Resistance Training on Muscle Mass, Neuromuscular and Physical Function in Older Adults: A Systematic Review and Meta-Analysis of Randomized Clinical Trials. J. Ageing Longev..

[95] Wei B.Z., Li L., Dong C.W., Tan C.C., Xu W. (2023). The Relationship of Omega-3 Fatty Acids with Dementia and Cognitive Decline: Evidence from Prospective Cohort Studies of Supplementation, Dietary Intake, and Blood Markers. Am. J. Clin. Nutr..

[96] Amlashi M.A., Payahoo A., Maskouni S.J., Dehghani E., Talandashti M.K., Ghelichi Y., Nikoumanesh M., Rezvani S., Shahinfar H., Shidfar F. (2025). Dose-dependent effects of omega-3 polyunsaturated fatty acids on C-reactive protein concentrations in cardiometabolic disorders: A dose–response meta-analysis of randomized clinical trials. Inflammopharmacology.

[97] Dwiputra B., Santoso A., Purwowiyoto B.S., Radi B., Pandhita B.A.W., Fatrin S., Ambari A.M. (2024). Current Evidence and Future Directions of Omega-3 Supplementation and Cardiovascular Disease Risk. Int. J. Angiol..

[98] Chang J.P.-C., Tseng P.-T., Zeng B.-S., Chang C.-H., Su H., Chou P.-H., Su K.-P. (2023). Safety of Supplementation of Omega-3 Polyunsaturated Fatty Acids: A Systematic Review and Meta-Analysis of Randomized Controlled Trials. Adv. Nutr..

[99] Richter C., Skulas-Ray A., Kris-Etherton P.M. (2016). Recommended Intake of Fish and Fish Oils Worldwide. Fish and Fish Oil in Health and Disease Prevention.

[100] Tocher D.R., Betancor M.B., Sprague M., Olsen R.E., Napier J.A. (2019). Omega-3 Long-Chain Polyunsaturated Fatty Acids, EPA and DHA: Bridging the Gap between Supply and Demand. Nutrients.

[101] Barros M.I., Brandão T., Irving S.C., Alves P., Gomes F., Correia M. (2025). Omega-3 Polyunsaturated Fatty Acids and Cognitive Decline in Adults with Non-Dementia or Mild Cognitive Impairment: An Overview of Systematic Reviews. Nutrients.

[102] Gutierres D., Pacheco R., Reis C.P. (2025). The Role of Omega-3 and Omega-6 Polyunsaturated Fatty Acid Supplementation in Human Health. Foods.

[103] Papanikolaou Y., Brooks J., Reider C., Fulgoni V.L. (2014). U.S. adults are not meeting recommended levels for fish and omega-3 fatty acid intake: Results of an analysis using observational data from NHANES 2003–2008. Nutr. J..

[104] Rondanelli M., Rigon C., Perna S., Gasparri C., Iannello G., Akber R., Alalwan T.A., Freije A.M. (2020). Novel Insights on Intake of Fish and Prevention of Sarcopenia: All Reasons for an Adequate Consumption. Nutrients.

[105] Cholewski M., Tomczykowa M., Tomczyk M. (2018). A Comprehensive Review of Chemistry, Sources and Bioavailability of Omega-3 Fatty Acids. Nutrients.

[106] Punia S., Sandhu K.S., Siroha A.K., Dhull S.B. (2019). Omega 3-metabolism, absorption, bioavailability and health benefits—A review. PharmaNutrition.

[107] Farková V., Křížová L., Dadáková K., Farka Z., Mascrez S., Eggermont D., Purcaro G., Kašparovský T. (2024). Changes in the fatty acid profiles and health indexes of bovine colostrum during the first days of lactation and their impact on human health. Food Chem..

[108] Skouvaklidou E., Theodoridis X., Tziona E., Vounotrypidis P., Dimitroulas T., Chourdakis M. (2024). Effectiveness of N-3 fatty acids supplementation on spondyloarthritis: A systematic review and meta-analysis. Clin. Nutr..

[109] Granato D., Zabetakis I., Koidis A. (2023). Sustainability, nutrition, and scientific advances of functional foods under the new EU and global legislation initiatives. J. Funct. Foods.

[110] Abubakr A. (2025). Improving rumen microbial efficiency in ruminal livestock: Current approaches and future insights—A review. Shendi Univ. J. Appl. Sci..

[111] Martucci M., Conte M., Bucci L., Giampieri E., Fabbri C., Palmas M.G., Izzi M., Salvioli S., Zambrini A.V., Orsi C. (2020). Twelve-Week Daily Consumption of ad hoc Fortified Milk with ω-3, D, and Group B Vitamins Has a Positive Impact on Inflammaging Parameters: A Randomized Cross-Over Trial. Nutrients.

[112] Feng J., Zhang Y., Zheng F., Cheng K., Zeng X., Li M., Zeng C., Chen X., Shen Q. (2025). The convergence of food science and nutrigenomics: Exploring new frontiers in innovation and development opportunities. Trends Food Sci. Technol..

[113] Almeida K.V., Resende T.L., Silva L.H.P., Dorich C.D., Pereira A.B.D., Soder K.J., Brito A.F. (2023). Feeding incremental amounts of ground flaxseed: Effects on diversity and relative abundance of ruminal microbiota and enteric methane emissions in lactating dairy cows. Transl. Anim. Sci..

[114] Newbold C.J., Ramos-Morales E. (2020). Review: Ruminal microbiome and microbial metabolome: Effects of diet and ruminant host. Animal.

[115] Zabor E.C., Kaizer A.M., Hobbs B.P. (2020). Randomized Controlled Trials. Chest.

