# Supplementary material for: Optimizing Omega-3 Polyunsaturated Fatty Acids for Healthy Ageing: Human Intake Evidence and Dairy Cow Dietary Interventions for Milk Enrichment
Source: Foods. 2026 Mar 19;15(6):1079. doi: 10.3390/foods15061079 (PMC13025747; doi:10.3390/foods15061079)
Supplement: Supplementary file 1 [file foods-15-01079-s001.zip › foods-4117053-supplementary.pdf]

Supplementary data

Table S1. PICOTS eligibility criteria of the study.

**P** (Population)	Adults (≥60 years old)
**I** (Intervention)	Implementation of delivery of diet/nutritional intervention with combination of omega-3 consumption.
**C** (Comparator)	Participants in the comparator group received in-person diet, nutritional advices or no intervention (CG).
**O** (Outcomes)	Cognitive and muscle mass and physical function and overall health such as cardio metabolic health and indexes (serum biomarkers of inflammation, lipid profile, glycose levels, blood pressure).<br>Body mass index, body weight, waist circumference, and functional capacity.
**T** (Time Factor)	Any intervention time period
**S** (Study design)	Randomized controlled trial (RCT)
